# Interspecies competition in oral biofilms mediated by *Streptococcus gordonii* extracellular deoxyribonuclease SsnA

**DOI:** 10.1038/s41522-022-00359-z

**Published:** 2022-12-12

**Authors:** Nadia Rostami, Robert C. Shields, Hannah J. Serrage, Catherine Lawler, Jane L. Brittan, Sufian Yassin, Halah Ahmed, Achim Treumann, Paul Thompson, Kevin J. Waldron, Angela H. Nobbs, Nicholas S. Jakubovics

**Affiliations:** 1 School of Dental Sciences, Faculty of Medical Sciences, Newcastle University, Newcastle, UK; 2 Department of Biological Sciences, Arkansas State University, Jonesboro, AR USA; 3 Bristol Dental School, University of Bristol, Bristol, UK; 4 Department of Restorative Sciences, University of Alabama at Birmingham, Birmingham, AL USA; 5 Protein and Proteome Analysis Facility, Faculty of Medical Sciences, Newcastle University, Newcastle, UK; 6 KBI Biopharma BV, Leuven, Belgium; 7 Biosciences Institute, Faculty of Medical Sciences, Newcastle University, Newcastle, UK

**Keywords:** Biofilms, Plaque

## Abstract

Extracellular DNA (eDNA) is a key component of many microbial biofilms including dental plaque. However, the roles of extracellular deoxyribonuclease (DNase) enzymes within biofilms are poorly understood. *Streptococcus gordonii* is a pioneer colonizer of dental plaque. Here, we identified and characterised SsnA, a cell wall-associated protein responsible for extracellular DNase activity of *S. gordonii*. The SsnA-mediated extracellular DNase activity of *S. gordonii* was suppressed following growth in sugars. SsnA was purified as a recombinant protein and shown to be inactive below pH 6.5. SsnA inhibited biofilm formation by *Streptococcus mutans* in a pH-dependent manner. Further, SsnA inhibited the growth of oral microcosm biofilms in human saliva. However, inhibition was ameliorated by the addition of sucrose. Together, these data indicate that *S. gordonii* SsnA plays a key role in interspecies competition within oral biofilms. Acidification of the medium through sugar catabolism could be a strategy for cariogenic species such as *S. mutans* to prevent SsnA-mediated exclusion from biofilms.

## Introduction

The oral cavity typically harbours between approximately 100 and 300 different bacterial taxa that colonise the surfaces of hard and soft tissues^1^. The taxonomic composition and distribution of bacteria within oral biofilms are determined by selective adherence processes, competitive and mutualistic interactions between species and the environment provided by the host^2–5^. The accumulation of dental plaque, the biofilm formed on the surfaces of teeth, occurs in a spatiotemporally ordered manner^6^. Pioneer colonisers capable of adhering to the acquired enamel pellicle lay the foundation of dental plaque and provide receptors for the recruitment of secondary colonisers, which eventually leads to the development of complex and dynamic microbial communities^7,8^. Pioneer colonizers are generally considered commensals or even beneficial for maintaining oral health^2,9,10^. However, if dental plaque is not properly controlled, dental caries or periodontitis can develop^11^. These two conditions are responsible for the vast majority of tooth loss worldwide^12^. Disease is associated with shifts in the composition and biochemical activities of dental plaque that result in a state of dysbiosis^13,14^. In the case of dental caries, excess frequency and/or amount of dietary sugars intake selects for dysbiotic biofilms enriched in aciduric and acidogenic bacteria that reduce the pH at the tooth surface and drive demineralization of tooth enamel or cementum. Further progression of the lesion results in destruction of the dentin and invasion of bacteria into the pulp chamber^15,16^.

Pioneer colonisers such as Mitis-group streptococci play important roles in controlling the overgrowth of more acidogenic and cariogenic bacteria such as *Streptococcus mutans*. For example, *Streptococcus gordonii* expresses an arginine deaminase (AD) system that counteracts the acidification of the biofilm by generating ammonia and carbon dioxide whilst producing energy for the cells^9,17–20^. In the presence of oxygen, *S. gordonii* and other members of the Mitis group of streptococci including *Streptococcus sanguinis* produce substantial (millimolar) amounts of hydrogen peroxide (H_2_O_2_), which can inhibit the growth of *S. mutans*^21^. In addition, it is possible that the pioneer colonisers compete with *S. mutans* for binding sites within the biofilm. *S. mutans* attachment is heavily dependent on the presence of sucrose, which is metabolised extracellularly to produce glucans that support adhesion and biofilm colonisation^22^. In the absence of sucrose, *S. mutans* adhesion appears to be mediated by extracellular DNA (eDNA)^23^. Extracellular DNA is also important for the structural integrity of mature *S. mutans* biofilms since treatment with DNase I results in biofilm dispersal^24,25^. It has recently been shown that eDNA is present in more complex biofilms including mixed-species dental plaque^26–31^. Treatment of dental plaque, particularly at the early stages of development, with exogenous DNase enzymes significantly reduces the biofilm biomass and selectively depletes certain species that appear to be particularly dependent upon eDNA^27,31,32^. In addition, eDNA serves as a nutrient source and reservoir for horizontal gene transfer within dental plaque^3,16^.

Many bacteria produce surface bound or secreted deoxyribonuclease (DNase) enzymes^33,34^. In biofilms, extracellular DNases serve a variety of functions including dissemination of bacterial cells, eDNA turnover and horizontal gene transfer^8,35–38^. In pathogens, extracellular DNase enzymes are often linked to virulence and evasion of host immune responses such as neutrophil extracellular traps (NETs)^39,40^. A number of oral bacteria including *S. gordonii* produce extracellular DNase enzymes^33,34,41^. However, their functions in biofilm maintenance and interspecies interactions are poorly understood. Here, we identified and characterised the enzyme responsible for extracellular DNase activity of *S. gordonii*. Furthermore, we investigated the effect of this enzyme on development of *S. mutans* biofilms or more complex mixed-species biofilms formed by human oral bacteria.

## Results

### Extracellular DNA in *S. gordonii* and *S. mutans* biofilms

**Fig. 1:**
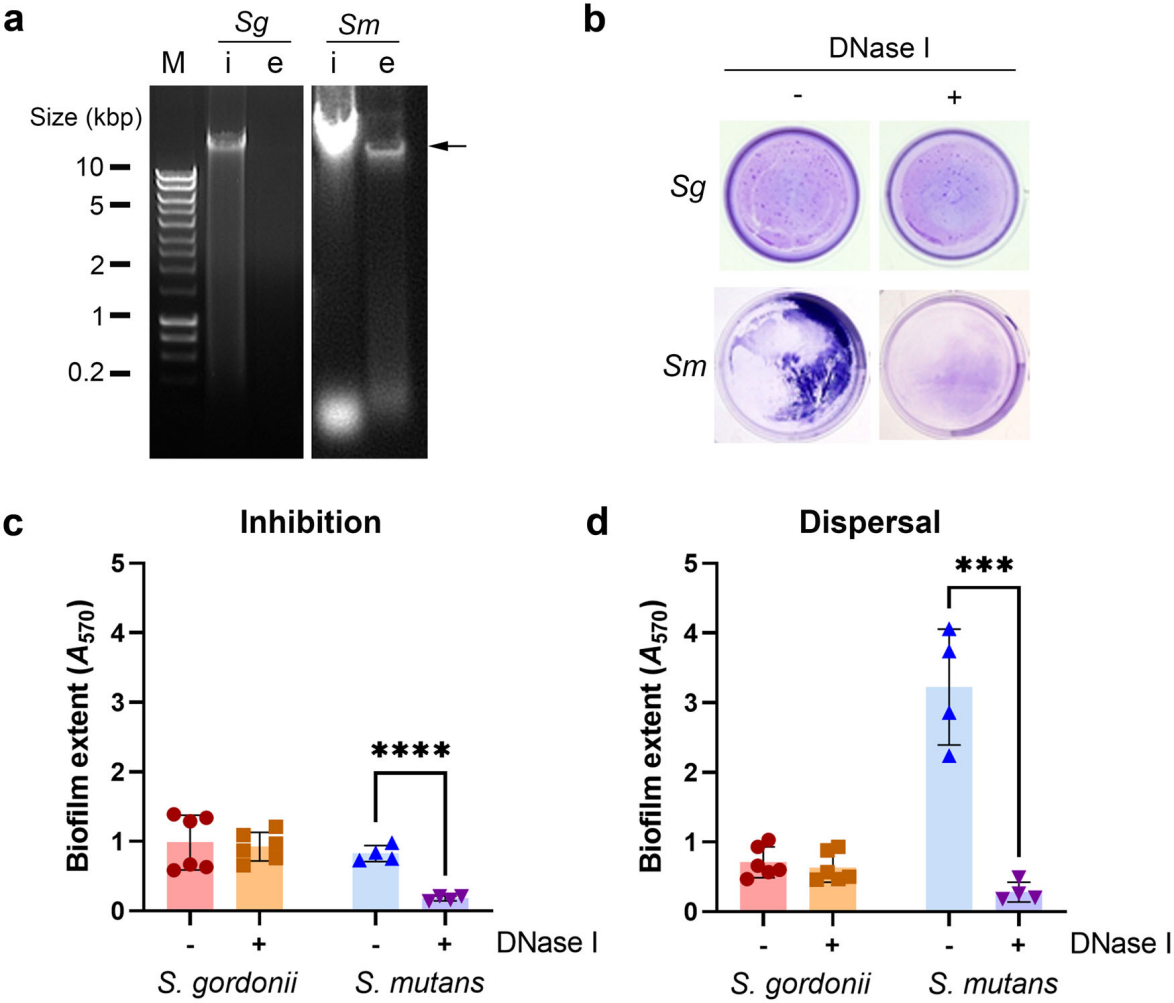
*S. mutans* but not *S. gordonii* forms biofilms in an eDNA-dependent manner. **a** Agarose gel electrophoresis of intracellular and extracellular DNA (iDNA and eDNA) isolated from *S. gordonii* and *S. mutans* biofilms. Biofilms were grown for 48 h in multi-well plates and cells were separated from extracellular matrix by centrifugation prior to DNA extraction. The black arrow indicates eDNA. Marker (M) was run on the same gel as the *S. gordonii* samples but separated from the sample lanes. *S. mutans* samples were run on a different gel. **b** Pre-established biofilms of *S. gordonii* and *S. mutans* grown for 20 h in BHY medium were treated with 5 μg/mL DNase I for 1 h at 37 °C. Biofilms were stained with crystal violet and biomass was quantified by dissolving the dye and measuring absorbance at 570 nm. **c** Quantification of crystal violet stained *S. gordonii* DL1 and *S. mutans* GS-5 biofilms grown in the presence and absence of 5 μg/mL DNase I. Biofilms were grown anaerobically for 20 h in BHY at 37 °C. *S. mutans* biofilms but not *S. gordonii* were inhibited when grown in the presence of 5 μg/mL DNase I. **d** Quantification of pre-formed *S. mutans* biofilms treated with 5 μg/mL DNase I for 1 h at 37 °C showed significant reduction by DNase I treatment (dispersal), whereas *S. gordonii* biofilms were not reduced. Data points are shown for 4–6 independent experiments, bars represent the mean and SD is indicated. The distribution of data passed the Shapiro-Wilk normality test and therefore statistical significance was calculated using Student’s t-test; ***p < 0.001, ****p < 0.0001.

To determine the extent of eDNA within the matrix of *S. gordonii* DL1 and *S. mutans* GS-5 biofilms, eDNA was extracted from monospecies biofilms grown statically for 48 h. A band at high molecular weight (>10 kbp) was observed in extracts isolated from *S. mutans* biofilms but not in *S. gordonii* biofilms (Fig. 1a). The size of the fragment was similar to intracellular chromosomal DNA (iDNA) extracted from *S. mutans* cells. This band was not observed following enzymatic treatment of the extracts with DNase I (Supplementary Fig. 1). NanoDrop spectrophotometry analysis of the extracts showed eDNA represented approximately 19% of the total DNA (iDNA + eDNA) in *S. mutans* biofilms while it accounted for only 6% of the total DNA in *S. gordonii* biofilms. The role of eDNA in maintaining the integrity of *S. mutans* and *S. gordonii* biofilms was investigated by treating pre-formed *S. mutans* and *S. gordonii* biofilms with 5 µg/mL DNase I for 1 h at 37 °C (Fig. 1b, c). Crystal violet staining revealed that DNase I treatment significantly reduced *S. mutans* biofilm biomass by >90%. By contrast, *S. gordonii* biofilms were not affected by DNase I treatment. Inclusion of DNase I during biofilm development significantly inhibited the accumulation of *S. mutans* biofilms but did not affect *S. gordonii* (Fig. 1d).

### *S. gordonii* expresses an extracellular DNase enzyme

**Fig. 2:**
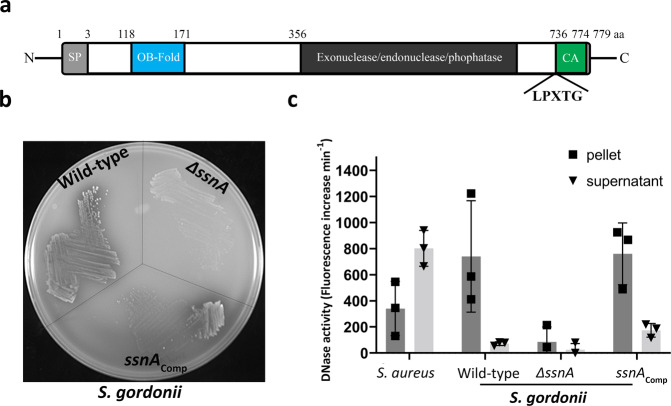
*ssnA* encodes the extracellular nuclease of *S. gordonii*. **a** Schematic diagram of the domain organisation of the protein encoded by the *ssnA* gene. Positions of predicted domains including a putative signal peptide (SP), an OB-fold, a nuclease domain and an LPxTG cell wall anchoring motif are marked with start and end amino acid residue numbers from the N-terminus. **b** Extracellular DNase activity was assessed using DNase Test agar. A clear halo around colonies of wild *S. gordonii* DL1 indicates DNase activity. No halo was visible in *S. gordonii* Δ*ssnA*, whereas extracellular DNase activity was restored in *S. gordonii ssnA*_Comp_. **c** The cell fraction and spent culture media were separated by centrifugation of planktonic cultures of wild type *S. gordonii*, the Δ*ssnA* mutant and *ssnA*_Comp_ strains, and the nuclease activity of each fraction was determined using a fluorescence based quantitative assay. *S. aureus* FH7 was included as a control. Extracellular DNase activity of *S. gordonii* was associated primarily with the cells, whereas *S. aureus* DNase activity was higher in conditioned medium. Identical volumes of cellular and supernatant fractions were used in analysis. Data points are shown for 3 independent repeats, bars represent the mean and SD is indicated.

We hypothesised that low levels of eDNA in *S. gordonii* biofilms may be due to the production of an extracellular DNase enzyme. Screening the *S. gordonii* DL1 genome using NCBI Blast identified three genes that potentially encoded proteins with DNase I-type domains. Of these, genes encoding an exonuclease III enzyme and a protein labelled RgfB did not have characteristics of secreted proteins. The third gene (SGO_RS08090; GenBank Accession WP_012130709.1) encoded a putative 779 amino acid protein with an N-terminal secretion signal and a C-terminal LPxTG motif for sortase-mediated anchoring to the cell wall, indicative of an extracellular cell wall-bound enzyme (Fig. 2a). In view of homology to other streptococcal extracellular nucleases including SsnA of *Streptococcus suis*^42^, the putative DNase gene of *S. gordonii* was termed *ssnA* (Streptococcal Surface Nuclease A). To test whether *ssnA* gene was responsible for extracellular DNase activity in *S. gordonii, ssnA* was knocked out and extracellular DNase activity was assessed using DNase Test agar (Fig. 2b). A zone of clearance was visible around colonies of wild-type *S. gordonii* indicating that these cells produced extracellular DNase. By contrast, no extracellular DNase activity was detected in the ∆*ssnA* mutant. Genetic complementation of the *ssnA* gene under its native promoter (*S. gordonii ssnA*_Comp_) restored the DNase activity (Fig. 2b).

To further explore the production of SsnA in *S. gordonii*, a quantitative fluorescence-based DNase activity assay was employed (Fig. 2c). DNase activity was determined in cell-bound fractions and supernatants. *Staphylococcus aureus* FH7, which is known to release DNase into the extracellular milieu, was included as a control. In wild-type *S. gordonii*, DNase activity was approximately 5-fold greater in the cell-associated fraction than spent medium, indicating that the majority of secreted SsnA remains bound to the cell wall. DNase activity was almost undetectable in the cell fraction and spent medium of *S. gordonii* Δ*ssnA*. Extracellular DNase activity was restored in the complemented strain and was mostly associated with the cellular fraction.

### Carbon catabolite repression (CCR) governs expression of SsnA

**Fig. 3:**
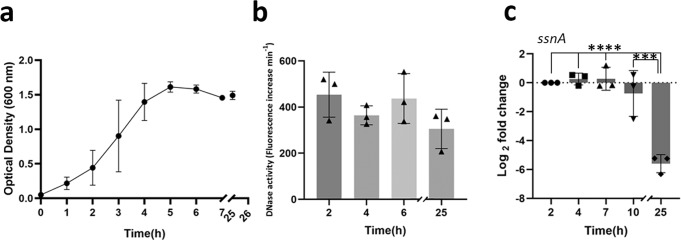
*ssnA* expression and SsnA activity during *S. gordonii* growth. **a** Growth curve of *S. gordonii* grown aerobically in BHY at 37 °C. **b** DNase activity of cells measured at 2, 4, 6, and 25 h using the quantitative fluorescent assay are shown. Optical density (OD_600 nm_) measurements were used for normalization. **c** RNA was extracted from the samples at different times through growth to evaluate *ssnA* expression using RT-qPCR. Fold change was normalised to 16 S rRNA gene expression. The result of 3 independent experiments and SE are shown for each assay. The distribution of data passed the Shapiro-Wilk normality test. Ordinary one-way ANOVA and Dunnett’s multiple comparisons were performed using GraphPad Prism 9 to determine statistical significance; ****p < 0.0001.

Initially, the expression and activity of SsnA was monitored through the growth of *S. gordonii* in BHY (Fig. 3a). No significant difference in cell-associated SsnA activity was detected between 2 h and 25 h (Fig. 3b). Gene expression of *ssnA* relative to expression of the 16 S rRNA gene was also stable during the exponential growth phase. However, expression decreased during stationary phase and was significantly reduced by >30-fold after 25 h (Fig. 3c).

Sequence analysis of the promoter region of *ssnA* revealed a consensus binding site for carbon catabolite protein A (CcpA), or possibly for the maltose regulator MalR protein (Supplementary Fig. 2a). To determine whether CcpA or MalR play a role in regulation of SsnA expression, ∆*ccpA* and ∆*malR* mutants were constructed and extracellular DNase activity was assessed in strains grown in the presence or absence of added sugars (Supplementary Fig. 2b). In Gram-positive bacteria such as *S. gordonii*, CCR is linked to uptake of sugars through the phosphoenolpyruvate-dependent sugar:phosphotransferase system (PTS). Substrate specificity is determined by the enzyme II (EII) component of the PTS. The *S. gordonii* EII^Man^ complex appears to have broad substrate specificity^43^ and we were unable to identify a sugar that is utilised by *S. gordonii* and that does not trigger CCR (Lin Zeng, personal communication). Purified SsnA was included as a control and degraded DNA in all conditions tested, as indicated by a zone of clearance on the agar. In wild type *S. gordonii*, DNA degradation was observed in the absence of added sugars or in galactose, but not in glucose, maltose or sucrose. Interestingly, DNase activity was observed in the ∆*ccpA* mutant grown in the presence or absence of any of the sugars tested (Supplementary Fig. 2). A genetically complemented strain, *S. gordonii ccpA*_Comp_, exhibited a similar pattern of DNase activity to the wild type. Deletion of *malR* did not affect DNase activity suggesting that this repressor is not involved in the regulation of *ssnA* expression under conditions tested here.

### Reduced pH from sugar fermentation is associated with inhibition of SsnA

**Fig. 4:**
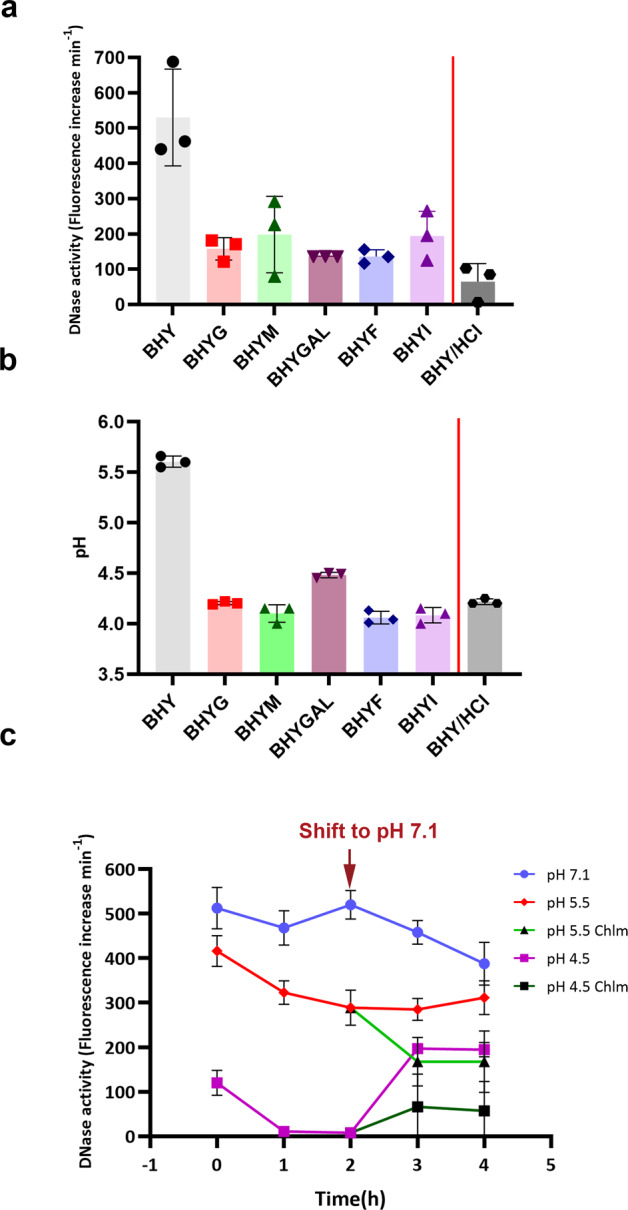
Sugar fermentation leads to acid inhibition of SsnA. **a** Extracellular nuclease activity of *S. gordonii* DL1 grown in BHY or BHY + 2% (w/v) sugars: glucose (BHYG), maltose (BHYM), galactose (BHYGal), fructose (BHYF), or inulin (BHYI). Extracellular DNase activity of *S. gordonii* cells grown in acidified medium BHY/HCl (BHY amended to pH 5.5 with HCl) is shown separated by a red line. Data points are shown for 3 independent repeats, bars represent the mean and SD is indicated. **b** The pH of cultures following growth to stationary phase in BHY, BHY + sugars or in BHY/HCl. **c** *S. gordonii* DL1 overnight cultures were harvested and re-suspended in buffer at pH 7.1 (blue line), 5.5 (red line) or 4.5 (magenta line). After 2 h (red arrow), cells were harvested and resuspended in PBS (pH 7.1) or PBS (pH 7.1) supplemented with chloramphenicol (Chlm; 12.5 μg/mL; light and dark green lines). DNase activity is shown for cell-associated SsnA. Data points are shown for 3 independent repeats, bars represent the mean and SD is indicated. Optical density (OD_600 nm_) measurements were used for normalization in each assay.

To quantify the extent of the sugar inhibition on SsnA activity, DNase activity of cells grown in BHY medium or BHY supplemented with different sugars was measured using a fluorescence-based assay (Fig. 4a). *S. gordonii* DNase activity was reduced by >50% in the presence of glucose, maltose, galactose, fructose or inulin. Streptococci produce acid from sugars and it was possible that the DNase enzyme activity may have been affected by the pH of the cultures. The pH of supernatants from stationary phase *S. gordonii* BHY cultures was approximately 5.6, whereas the pH of cultures grown with galactose was approximately 4.6 (Fig. 4b). The pH was further reduced to between 4 and 4.2 in cultures grown with glucose, maltose, fructose or inulin. To assess the impact of low pH on DNase activity, BHY was amended to pH 5.5 by the addition of HCl and used to culture *S. gordonii* cells to stationary phase. The final pH following growth was approximately 4.2 and almost no DNase activity was detected in cells grown in acidified BHY (Fig. 4).

We hypothesised that the effects of low pH on SsnA activity were not directly due to enzyme inhibition since the assay was performed after washing cells and resuspending in PBS at pH 7.1. To further explore pH-mediated reduction of SsnA activity, *S. gordonii* DL1 was incubated in neutral or acidic buffers and transferred to pH 7.1 in the presence or absence of the protein synthesis inhibitor chloramphenicol (Fig. 4c). The total DNase activity was lower in cells incubated at pH 4.5 or 5.5 than in cells incubated at pH 7.1. Following a shift to pH 7.1, SsnA activity increased in cells that had been incubated in pH 4.5 buffer. The recovery of SsnA activity was impaired in the presence of chloramphenicol indicating that *de novo* protein synthesis was required to restore enzyme activity. In cells that had been incubated for 2 h at pH 5.5, SsnA activity increased slightly, but not significantly, following a shift to pH 7.1. In the presence of chloramphenicol, SsnA activity continued to decrease after shifting to pH 7.1 indicating that active enzyme was not being restored.

### pH and metal dependence of purified SsnA

**Fig. 5:**
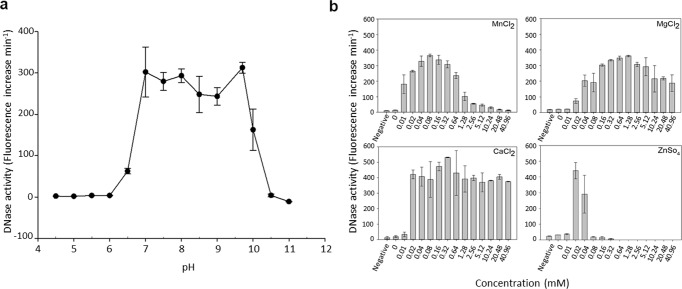
pH and metal ion requirements of SsnA. **a** The nuclease activity of purified SsnA was quantified at pH ranging from 4.5 to 11. **b** Using the quantitative fluorescent assay, the DNase activity of SsnA was tested in the presence of a range of Mn^2+^, Ca^2+^, Mg^2+^ and Zn^2+^ concentrations. Negative controls without enzyme are indicated. In all panels, points or bars indicate means from 3 independent experiments and SE is shown.

To characterise the enzyme activity in more detail, SsnA was purified as a GST-tagged construct. The identity of the purified construct was confirmed by peptide mass fingerprinting (Supplementary Fig. 3). In preliminary experiments, the GST tag was cleaved with thrombin and removed by affinity purification. The enzyme was shown to be active with or without the GST tag (Supplementary Fig. 4). Since removal of the tag resulted in a significant loss of yield of the protein, we used the GST-tagged construct for subsequent experiments. Initially, the effects of pH on DNase activity were investigated (Fig. 5a). The optimum pH was between 7 and 9.5. No activity was detectable below pH 6.5 or above pH 10.5.

Nucleases are frequently dependent on a divalent metal cofactor^44^. Inductively coupled plasma mass spectrometry (ICP-MS) analysis of SsnA did not detect any metal ions associated with the purified protein. To determine the preferred metal co-factors for SsnA, the enzymatic activity of SsnA was tested in the presence of Mn^2+^, Ca^2+^, Mg^2+^ or Zn^2+^ (Fig. 5b). In the absence of added metals, no DNase activity was detected in SsnA preparations. Addition of 10 µM MnCl_2_, 20 µM CaCl_2_, 40 µM MgCl_2_, or 40 µM ZnSO_4_ resulted in high levels of DNase activity. Increasing the concentration of ZnSO_4_ to 80 µM led to almost complete inactivation of DNase. DNase activity was also abrogated in high concentrations of MnCl_2_ (>1.28 mM MnCl_2_).

### SsnA disperses *S. mutans* biofilms under neutral but not acidic conditions

**Fig. 6:**
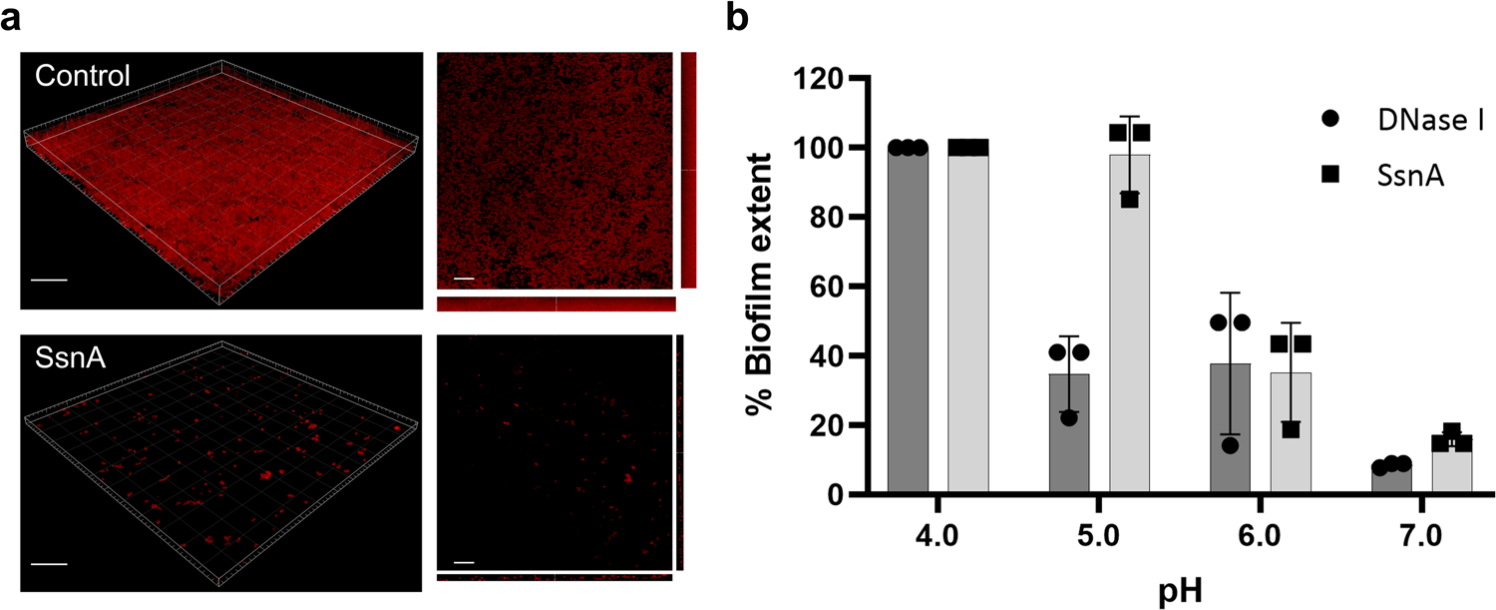
SsnA disperses pre-established *S. mutans* biofilm in a pH dependent manner. **a** Confocal laser scanning microscopy of pre-established 20 h biofilms of *S. mutans* treated with PBS (control) or 5 µg/mL SsnA for 1 h at 37 °C. Propidium iodide (20 μM) was used to visualise the cells. Scale bars are 30 µm (left panels) or 20 µm (right panels). **b** Biomass of pre-established *S. mutans* biofilms treated with DNase I (5 µg/mL) or SsnA (5 µg/mL) at pH 4, 5, 6, and 7. Crystal violet assay was used for quantification. Data points are shown for 3 independent repeats, bars represent the mean and SD is indicated.

As shown above, *S. mutans* GS-5 biofilms were sensitive to treatment with DNase I whereas *S. gordonii* biofilms were not. We hypothesised that SsnA would also disperse pre-formed *S. mutans* biofilms. Using confocal laser scanning microscopy, *S. mutans* biofilms were almost completely eliminated by treatment with SsnA (Fig. 6a). Inhibition was found to be dependent on the prevailing pH (Fig. 6b). At pH 4 or 5, biofilms were not removed by SsnA. By contrast, *S. mutans* biofilms were reduced to <40% of the control biomass (without enzyme treatment) by DNase I at pH 5. At pH 6, both DNase I and SsnA were effective at reducing *S. mutans* biofilms and biomass was reduced to <40% of the controls. Biofilm removal was even greater at pH 7, and only 10–20% of the biofilm remained following treatment with DNase I or SsnA. Based on these findings we hypothesised that *S. gordonii* wild-type cells, which express SsnA, could disperse *S. mutans* GS-5 biofilms. To test this hypothesis, pre-established *S. mutans* biofilms grown for 20 h were treated with *S. gordonii* wild-type or Δ*ssnA* cells or with spent culture medium (Supplementary Fig. 5). Treatment with wild-type *S. gordonii* cells or culture supernatant significantly reduced the *S. mutans* biofilm biomass to approximately 60% of the control. By contrast, treatment with *S. gordonii* Δ*ssnA* cells did not reduce *S. mutans* biofilms. A small, but significant reduction in *S. mutans* biofilms was observed following treatment with *S. gordonii* Δ*ssnA* culture supernatant (Supplementary Fig. 5). Overall, these data show that SsnA promotes the removal of *S. mutans* biofilms by *S. gordonii*.

### SsnA inhibits the accumulation of oral microcosm biofilms

**Fig. 7:**
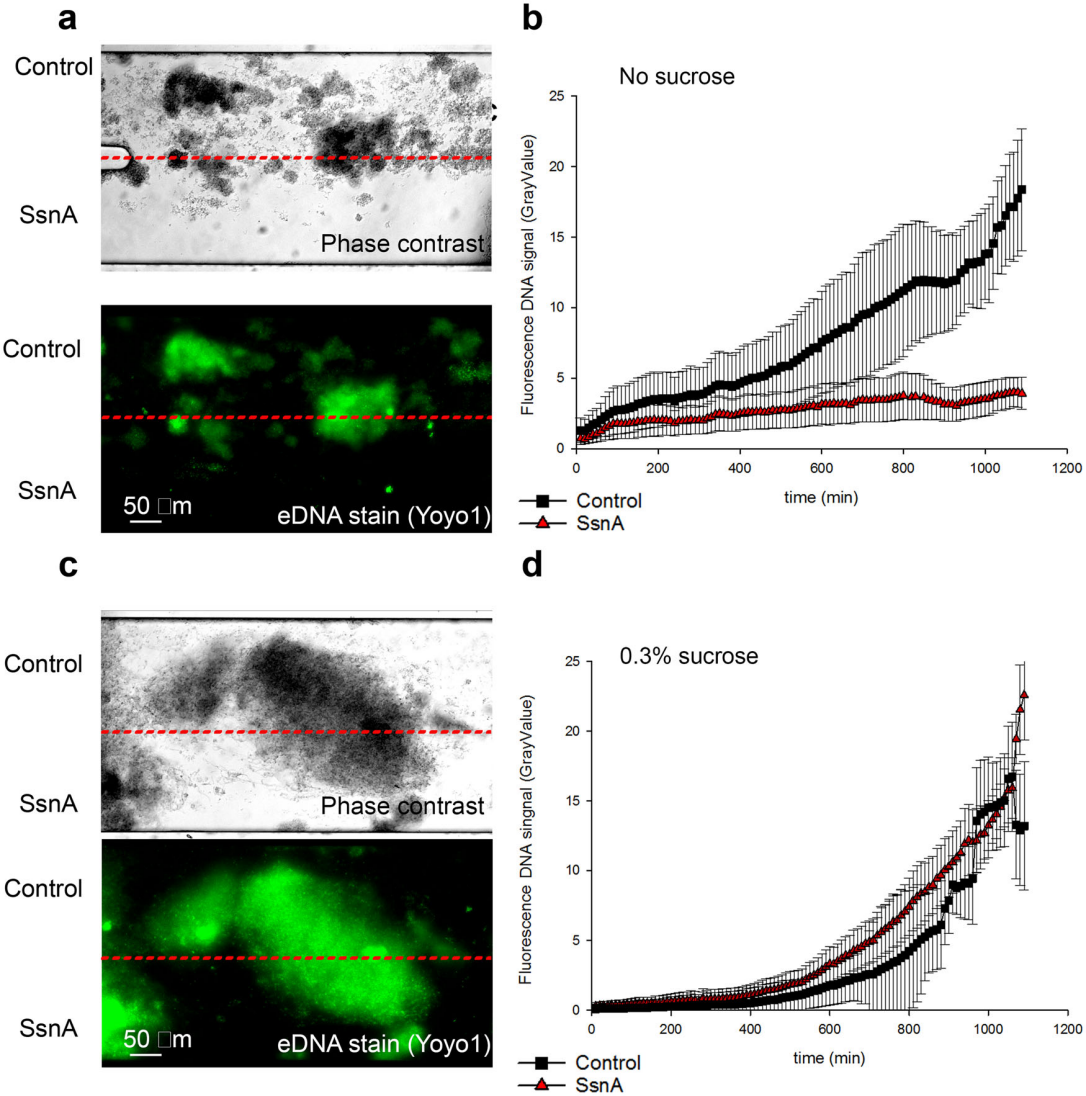
SsnA prevents accumulation of oral microcosm biofilms grown under flow of natural human saliva. **a** Visualisation of oral microcosm biofilms developed in the channel of BioFlux dual inlet microfluidic system with saliva containing SsnA (5 µg/mL) feeding the bottom half of the channel. Microcosm biofilms were grown for 20 h. Yoyo-1 (2.4 nM) was used to stain eDNA within the biofilm. **b** Quantification of eDNA accumulation for the SsnA-containing and SsnA-free section of the growth channel over 20 h. Images were taken every 10 min and eDNA amounts were determined using GrayValue within FIJI software. **c** Visualisation of microcosm biofilms grown in 0.3% sucrose-supplemented natural saliva. SsnA (5 µg/mL) was included in the reservoir feeding the bottom half of the channel. Yoyo-1 (2.4 nM) was included for eDNA staining. **d** Quantification of eDNA accumulation for the SsnA-containing and SsnA-free section of the growth channel over 20 h of growth in saliva supplemented with 0.3% sucrose. Images were taken every 10 min and eDNA amounts were determined using GrayValue within FIJI software. Mean and SE from 3 independent repeats are shown. Scale bars are 50 µm.

To determine whether SsnA can control the development of more complex oral biofilms, we employed a dual inlet BioFlux microfluidic system to grow oral microcosm biofilms in the presence or absence of SsnA under the flow of natural saliva as the sole nutrient source. The system was inoculated with unsterilised saliva to seed the channels. Biofilms were cultured for 20 h and images were taken every 10 min (Fig. 7a; Supplementary Movie 1). A clear inhibition of oral microcosm development was observed over the 20 h of growth in the half of the channel in which SsnA was included. Staining of extracellular DNA (eDNA) with Yoyo-1 showed that eDNA was also markedly reduced in the presence of SsnA. The fluorescence signal was quantified over the course of biofilm formation (Fig. 7b). A consistent increase in eDNA levels was observed over 20 h of biofilm development in the top half of the channel (control) whilst in the lower half (SsnA-supplemented), levels of eDNA remained stable throughout the experiment.

### Sucrose fermentation blocks eDNA degradation by SsnA in oral microcosm biofilms

**Fig. 8:**
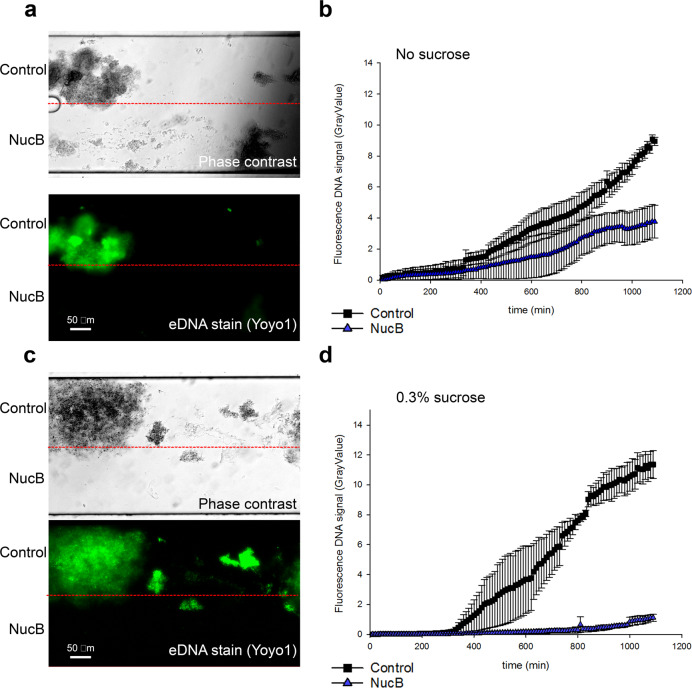
NucB exerts antibiofilm activity in the presence or absence of sucrose. **a** Oral microcosm biofilms grown for 20 h in natural saliva and supplemented with NucB in the lower of the microfluidic channel. Yoyo-1 (2.4 nM) dye was used for eDNA visualisation. **b** eDNA quantified in the two halves of the growth channel over 20 h of growth. eDNA was measured through GrayValue determination in FIJI software for images taken every 10 min. **c** Sucrose (0.3%) supplemented saliva was used to grow microcosm biofilms and NucB was included in the lower half of the growth channel. **d** eDNA quantified for the NucB-containing and NucB-free halves of the growth channel over 20 h of growth in sucrose (0.3%) containing saliva. Mean and SE from 3 independent repeats are shown. Scale bars are 50 µm.

Previous work had shown that sugars lead to a reduction in pH and suppression of SsnA enzyme activity (Fig. 4). To assess the effects of sugar on the control of oral microcosm biofilms by SsnA, biofilms were cultured in the BioFlux system in saliva supplemented with 0.3% sucrose (Fig. 7c; Supplementary Movie 2). Under these conditions, no reduction in biofilm accumulation was observed in the presence of SsnA (Fig. 7c, d). To further explore the impact of sucrose on eDNA in oral microcosm biofilms, *Bacillus licheniformis* extracellular nuclease NucB was employed since it has previously been shown to control the development of oral microcosm biofilms under static conditions^31^. In the presence of NucB, biofilm development in saliva was strongly inhibited compared with controls (Fig. 8a, b; Supplementary Movie 3). Similar inhibition was observed in cultures grown in saliva supplemented with 0.3% sucrose (Fig. 8c, d; Supplementary Movie 4). Taken together, the above data indicate that eDNA is important for the development of oral microcosm biofilms and that SsnA is not effective at controlling eDNA-dependent biofilm accumulation in the presence of sucrose.

## Discussion

Here, we identified and characterized SsnA, an extracellular cell wall-associated DNase enzyme produced by *S. gordonii*. Extracellular DNase enzymes are widely produced by pathogenic species of streptococci and are important virulence factors^39,45–47^. A number of oral commensal streptococci including *S. sanguinis*, *Streptococcus intermedius*, *Streptococcus oralis*, *Streptococcus parasanguinis* and *Streptococcus mitis* produce detectable levels of extracellular DNase activity^34,41^. Of these, only the extracellular DNase of *S. sanguinis* has been characterised. This enzyme, termed SWAN (Streptococcal Wall-Anchored Nuclease), was shown to mediate escape from neutrophil extracellular traps (NETs)^41^. There is evidence that *S. mutans* can also escape from NETs using a deoxyribose aldolase DeoC^48^. However, extracellular nuclease production by *S. mutans* is weak or undetectable in DNase activity assays^34^. Our data have identified a role for *S. gordonii* SsnA in modulating biofilm formation by competing species such as *S. mutans*. Further, it was shown that the ability of SsnA to mediate interspecies competition within biofilms is dependent upon the prevailing pH and concentration of sugars. Therefore, it appears that streptococcal extracellular DNases may play important roles in maintaining the stability of dental plaque biofilms.

SsnA was identified by searching the *S. gordonii* genome for genes encoding proteins with DNase I-like domains. The DNase I family includes a diverse range of nucleases and phosphatases that share a conserved protein fold^49^. Although our analysis suggests that the majority of SsnA is cell-bound, a mass spectrometry study of the whole secretome of *S. gordonii* revealed the presence of SsnA (SGO_1651) amongst 107 secreted proteins^50^. Therefore, it is likely that a proportion of the enzyme is released from the cell surface. Homologues of *S. gordonii* SsnA are present in a wide range of oral streptococci including *S. sanguinis*, *S. intermedius*, *S. constellatus*, *S. anginosus* and *S. parasanguinis* (Supplementary Fig. 6), indicating that the enzyme likely plays important roles in oral biofilms. In addition, more distantly related homologues are present in pathogenic streptococci including *S. pyogenes* and the important animal pathogens *S. suis*, *S. equi* and *S. iniae* (Supplementary Fig. 6).

The SsnA activity was strongly reduced in the presence of sugars. Supplementation with glucose or the glucose-containing disaccharides led to complete inhibition of extracellular DNase activity. Upstream of the *ssnA* gene is a CRE sequence motif and regulation was abrogated in a Δ*ccpA* mutant. Hence, it is likely that CcpA acts at the level of gene regulation to control the expression of *ssnA* in the presence of glucose. Unfortunately, the Δ*ccpA* mutant grew slowly in broth cultures and we were unable to obtain reproducible data from RT-qPCR analysis of *ssnA* expression in this strain. The regulation of extracellular DNase enzymes by CcpA has been observed in other streptococcal species including *S. suis* and *S. pyogenes*^51,52^. In *S. gordonii*, CCR is elicited by a broad range of sugars and we were unable to identify a sugar that does not lead to CCR in *S. gordonii*^43^. In fact, some sugars such as galactose and lactose elicit complex regulatory responses in *S. gordonii* that involve multiple regulators in addition to CcpA^53^. It is interesting that galactose did not reduce SsnA activity in an agar plate assay (Supplementary Fig. 2), whereas it strongly repressed SsnA in broth cultures (Fig. 3). We hypothesise that this may be due to regulation pathways that compensate for galacatose-driven CcpA-mediated repression of *ssnA* expression on agar plate cultures. Further work will be needed to identify the regulatory pathways that control *ssnA* expression in biofilms.

The activity of SsnA may have also been affected by the pH in the growth medium. Sugar metabolism led to an acidification of the growth medium to pH < 5.0. Incubating cells at similarly low pH led to a rapid loss of extracellular DNase activity that was only restored through de novo protein synthesis. Mammalian DNase I enzymes tend to have a relatively low pH optimum, around 6.5–7, whereas other DNase I family members show a preference for higher pH conditions^54^. The pH activity range of recombinant SsnA (6.5 to 10.5) appears to be slightly higher than the *S. sanguinis* homologue SWAN, which is active from pH 5.5 to 9.0, with maximal activity at approximately neutral pH^41^. Incubation of recombinant SsnA in low-pH conditions did not lead to irreversible inactivation of the enzyme, and activity was fully restored when the enzyme was switched to a higher-pH buffer (Supplementary Fig. 7). Therefore, it appears that the inactivation of cell wall-bound SsnA following growth in sugars or low-pH medium is primarily due to protein turnover rather than direct inhibition of the enzyme. Nevertheless, it should be noted that chloramphenicol will result in the broad shutdown of protein synthesis, which could indirectly impact SsnA activity, for example by influencing the pH within cells and in the immediate environment.

In addition to pH, metal ions are important for the activity of DNase I family enzymes. For example, the activity of *S. sanguinis* SWAN is enhanced by Mg^2+^ and Ca^2+^^41^. Similarly, we found that either Mg^2+^ or Ca^2+^ promoted SsnA activity. This suggests that SsnA can utilise either of these metals, rather than requiring both like SWAN or some other DNase I-family enzymes. At low concentrations, Mn^2+^ and Zn^2+^ also appeared to activate SsnA. However, the activity markedly decreased at >64 μM Mn^2+^ or >40 μM Zn^2+^. Low concentrations (40 μM) of the chelating agent EDTA were included in these experiments to remove trace metals associated with the enzyme or DNA substrate. It is possible that Zn^2+^ displaced Mg^2+^ or Ca^2+^ from the EDTA, indirectly activating the enzyme. At higher concentrations, Zn^2+^ in particular appears to be a potent inhibitor of SsnA. This is consistent with other DNase I family enzymes including human DNase I that are inhibited by Zn^2+^^55^. On the other hand, we speculate that the decrease in the activity at higher Mn^2+^ concentrations may be caused by significant binding of Mn^2+^ to the DNA substrate, interfering with the enzyme’s recognition/interaction. Concentrations of Zn^2+^ in saliva are in the order of 0.2–1.2 μM, whereas Ca^2+^ is present at approximately 0.5–2.75 mM and Mg^2+^ at 7–30 μM^56^. These concentrations would potentially support activity of SsnA in oral biofilms.

The role of extracellular DNase enzymes in mediating interspecies interactions in streptococcal biofilms has not previously been investigated. Here, we showed that biofilms formed by *S. mutans* GS-5 are dependent on eDNA for stability. When cultured with NucB DNase enzyme, *S. mutans* NG8 produced approximately 20% of the biofilm biomass compared with control (NucB absent), whereas *S. mutans* UA159 was only reduced to approximately 80% of the control biomass (Supplementary Fig. 8), a level that is consistent with other published work^24^. It is not clear why some strains are more dependent on eDNA than others, although this phenomenon has also been observed for other oral streptococci^57^. It should be noted that some laboratory cultures of *S. mutans* GS-5, including our own, carry mutations in *pac* and *gbpC* genes, which lead to secretion of the cell surface protein PAc (antigen I/II) and a lack of production of GbpC (glucan-binding protein C)^58^. Future studies are needed to determine whether PAc and/or GbpC affect the dependence of *S. mutans* biofilms on eDNA.

The ability of SsnA to disperse static *S. mutans* GS-5 biofilms was dependent on the pH of the medium. At pH 5.0, SsnA did not disperse biofilms in contrast to DNase I, which retained anti-biofilm activity. Interestingly, at pH 6.0, SsnA and DNase I reduced biofilms to approximately 40% of levels in controls, indicating that both enzymes were active in these conditions. By contrast, recombinant SsnA was not active below pH 6.5 when tested against a fluorescent oligonucleotide probe. It is possible that the environment within biofilms may buffer the low pH^59^. Alternatively, this could be due to differences in the nature of the DNA substrate. The ability of SsnA to control *S. mutans* biofilms at approximately pH 6.0 may be important in conditions within the oral cavity since it has been shown that *S. mutans* biofilms contain less glucan and are more dependent on eDNA when cultured at pH 6.0 than when grown at pH 7.0^60^. Nevertheless, reduction of pH to 5.0 or lower may represent a defensive strategy of *S. mutans* against SsnA. It is noteworthy that sucrose and starch availability stimulate further eDNA release and acid production by *S. mutans* leading to establishment of a stable biofilm^25^. Consistently, growth of *S. mutans* in mixed-species biofilms that also contain *S. gordonii* biofilm is enhanced in the presence of sucrose^61,62^.

The presence of SsnA inhibited growth of mixed-species oral microcosm biofilms under flow of natural human saliva, suggesting that the anti-biofilm activity of SsnA is not specific to *S. mutans* biofilms. In addition to *S. mutans*, single species biofilms of several oral microorganisms such as *S. intermedius* and *S. sanguinis* have been shown to be sensitive to treatment with DNase enzymes^63–65^. Since oral biofilm development is strongly affected by interspecies cell-cell interactions^66^, we speculate that exclusion of certain species could have dramatic effects on the accumulation of dental plaque. The deleterious effects of SsnA on mixed-species oral biofilms are consistent with results obtained from treatment of in situ dental plaque grown with DNase I^32^. Previously, we showed that the presence of NucB DNase during oral microcosm development leads to reduced biomass^67^. Intriguingly, the microbial diversity in oral biofilms was also affected. The biofilm developed in presence of NucB contained reduced proportions of anaerobic and proteolytic late coloniser species such as *Peptostreptococcus*, *Porphyromonas*, and *Prevotella*^31^. Further work is required to establish whether SsnA selectively inhibits certain species within the biofilm.

Based on our findings, we speculate that it may be possible to target the expression of streptococcal DNases such as SsnA to control the growth of dental plaque. However, the inhibition of SsnA by low pH may restrict the development of this strategy against cariogenic biofilms. We note that SsnA was not effective at controlling biofilm growth in sucrose-supplemented saliva, possibly due to the production of acid by microorganisms within the biofilm. The production of glucans which can interact with DNA and stabilise the matrix^68^ may also have contributed to the lack of activity of SsnA in these conditions. Further studies aiming at understanding the regulatory mechanisms governing the production and the activity of SsnA, and effects of oral microbial extracellular DNases on the structure and microbial composition of dental plaque, could lead to development of novel approaches for controlling multispecies biofilms by targeting the extracellular matrix.

## Methods

### Bacterial strains and growth conditions

*Streptococcus gordonii* DL1 (Challis), *Staphylococcus aureus* FH7 and *Streptococcus mutans* GS-5 (Supplementary Table 1) were routinely cultured in BHY medium (Brain Heart Infusion 37 g/L and 5 g/L yeast extract) at 37 °C anaerobically (10% CO_2_, 10% H_2_ and 80% N_2_ in a Ruskinn BugBox Plus, Ruskinn Technology Ltd, Bridgend, UK). BHY agar (BHY medium plus 15 g/L Bacto-agar) was used as solid medium. When necessary, antibiotics were included as follows: kanamycin (250 μg/mL), spectinomycin (250 μg/mL) and erythromycin (10 µg/mL for chromosomal insertions and 2 µg/mL for plasmids). *Escherichia coli* was cultured in Lysogeny Broth (Melford Laboratories, Ipswich, UK) at 37 °C with shaking at 200 rpm.

### Intracellular and extracellular DNA extraction

Intracellular DNA (iDNA) or extracellular DNA (eDNA) was extracted as described by Kreth et al.^21^. *S. gordonii* or *S. mutans* were cultured overnight in TYEG medium containing 10 g Bacto tryptone, 5 g yeast extract, 3 g K_2_HPO_4_ and 2 g glucose per L, adjusted to pH 7.5. A total of 10 µL were transferred to 2 mL fresh TYEG in wells of a 12-well plastic dish and cultures were incubated with gentle rocking (20 rpm) for 72 h with changes of medium after each 24 h. The supernatant was aspirated and wells were washed twice with 1 mL phosphate buffered saline (PBS), pH 7.4. After aspirating the liquid, a further 1.5 mL PBS was added to one well and the biofilm was harvested by gentle scraping with a tissue culture scraper. To provide sufficient biomass for DNA extraction, this sample was transferred to an equivalent well and additional biofilm was scraped. In total, biofilms from four wells were combined for each sample. Cells were harvested by centrifugation at 12,000 *g*, 4 °C, 30 min and the supernatant (eDNA sample) and pellet (containing cells with iDNA) were stored at −20 °C until further purification.

For purification of eDNA, an equal volume of 25:24:1 phenol:chloroform:isoamyl alcohol was added and mixed by inversion 30–40 times. Following centrifugation at 16,000 *g*, 4 °C for 5 min, the aqueous phase was collected and the phenol:chloroform:isoamyl alcohol extraction was repeated. After another centrifugation, the aqueous phase was collected and DNA was precipitated by adding one tenth volume 3 M sodium acetate, pH 5.2 and mixing. Two-thirds volume of isopropanol was added and mixed, and eDNA was pelleted by centrifugation at 16,000 *g*, 4 °C for 10 min. After washing with 1 ml 100% ethanol, the pellet was air dried and resuspended in 20 µL 10 mM Tris-HCl, pH 8.0.

To extract iDNA, cell pellets were resuspended in 150 µL spheroplasting buffer (20 mM Tris-HCl, 10 mM MgCl_2_ and 26% (w/v) raffinose, adjusted to pH 6.8). To this, 1.5 µL lysozyme from a 250 µg mL^−1^ stock and 5 µL mutanolysin from a 10,000 U mL^−1^ stock were added and samples were incubated for 30 min at 37 °C. Cells were lysed by addition of 150 µL T&C Lysis solution containing 1 µL proteinase K (Epicentre MasterPure DNA purification kit, Epicentre Biotechnologies, Madison, WI, USA) and DNA was extracted by following the manufacturer’s instructions.

### Identification of putative DNase I family proteins and *ssnA* promoter region

To identify potential DNase I family homologues in *S. gordonii*, the DNase I domain superfamily was identified in the Superfamily database (https://supfam.mrc-lmb.cam.ac.uk/SUPERFAMILY/cgi-bin/scop.cgi?sunid=56219). *S. gordonii* DNase I family members were identified under ‘genome assignments’. The *ssnA* gene and promoter region was retrieved from the *S. gordonii* Challis genome (GenBank accession CP000725.1). Putative transcription factor binding sites were identified using PePPER^69^.

### Biofilm inhibition and dispersal assay

Bacteria were inoculated in 200 μL of media in triplicate in polystyrene 96-well plates. Plates were incubated anaerobically at 37 °C for 20 h. When performing biofilm inhibition assays, bovine DNase I or GST-SsnA (5 µg/mL) was included at the point of inoculation. For dispersal assays, enzymes (5 µg/mL) were added to 20 h old biofilms and incubated for a further hour at 37 °C. Biofilm extent was quantified using the crystal violet assay^57^. Biofilm assays were repeated three times independently. The significance of differences between absorbance readings was determined by Student’s two sample t-test.

### Fluorescence based quantitative DNase activity assay

An oligonucleotide probe, 5′ HEX-CCC CGG ATC CAC CCC- BHQ2 3′ (PrimeTime probe Integrated DNA Technologies), was used for quantification of DNase activity as described by Kiedrowski et al.^70^. The probe (2 μM of the probe in a buffer consisting of 20 mM Tris-HCl pH 8) was added to 25 μL of a nuclease source (cells or purified enzyme) in a well of a 384-well microtiter plate (Greiner Bio-One, Stonehouse, UK) and the rate of fluorescence change (λ_ex_: 530 nm, λ_em_: 590 nm) was measured at 37 °C over 30 min using the Synergy HT (BioTek, Swindon, UK) microplate reader.

### Mutagenesis and genetic complementation of *S. gordonii* DL1

Disruption of *ssnA* was carried out by replacing the chromosomal *ssnA* with the *aad9* spectinomycin resistance determinant. Flanking regions of the *ssnA* gene were PCR amplified using primers SsnAF1 and SsnAR1 to generate a 484-bp product in the 5′ region of *ssnA* and SsnAF2 and SsnAR2 to generate a 674-bp product in the 3′ end of *ssnA*. The *aad9* gene (782-bp) was amplified from plasmid pFW5 (Supplementary Table 1) with primers aad9_SsnAF and aad9_SsnAR primers. These PCR reactions were performed with Taq Reddymix (Sigma Aldrich, Gillingham, UK) and cycling conditions of 94 °C, 2 min, 35 cycles of 94 °C for 10 s, 56 °C for 30 s, 68 °C for 90 s, then 68 °C for 7 min and 4 °C hold. The PCR products were mixed in equimolar ratios and amplified in an overlap extension PCR using primers SsnAF1 and SsnAR2 with the Expand Long Range PCR enzyme mix (Sigma Aldrich). Thermocycling was performed as follows: 92 °C, 2 min, 10 cycles of 92 °C, 10 s, 56 °C, 15 s, 68 °C 150 s, 25 cycles of 92 °C, 10 s, 56 °C, 15 s, 68 °C 150 s and increasing by 20 s per cycle, then 68 °C for 7 min and 4 °C hold. The resulting product was used for transformation of *S. gordonii* DL1. For this, *S. gordonii* was cultured in a candle jar to early exponential phase (OD_600 nm_ ~ 0.3) in BHY medium supplemented with 1 µL mL^-1^ fetal calf serum and 0.1% (w/v) glucose (BHY/FCS/G). After a further 60 min at 37 °C, cultures were dispensed in 0.8 mL aliquots and approximately 1 µg of the PCR product was added. After incubation for a further 4 h, cultures were diluted and cultured on solidified BHY medium for 36 h in a candle jar. Successful disruption and replacement of *ssnA* gene with the *aad9* gene was confirmed by DNA sequencing.

Disruption of *ccpA* was carried out using a similar approach by replacing *ccpA* with the *kanR* kanamycin resistance gene. The upstream region of *ccpA* was amplified with CcpAF1 and CcpAR1 generating a 520-bp fragment and the downstream region of *ccpA* was amplified using CcpAF2 and CcpAR2. The *kanR* gene was amplified with KanR_F and KanR_R primers using pK18 vector (Supplementary Table 1) as template. Gibson Assembly Master Mix (New England BioLabs, Ipswich, Massachusetts, USA) was employed for fusion of the upstream and downstream fragments of *ccpA* to the *kanR* gene according to the instructions of the manufacturer. The resulting 1,795-bp fragment was used for transformation of *S. gordonii* cells. Successful disruption and replacement of the *ccpA* gene with the *kanR* gene was confirmed by DNA sequencing.

Mutagenesis of the *malR* gene was achieved by replacing *malR* with the *ermAM* erythromycin resistance cassette. Using the primers malRF1 and malRR1, a 471-bp fragment upstream of *malR* gene was amplified. malRF2 and malRR2 were used to amplify a 421-bp sequence downstream region of *malR* gene. The malRR1 and malRF2 primers contained 17-bp complementary sequences for ermAMF2 and ermAMR2 primers. After amplifying the *ermAM* cassette from pVA838 (Supplementary Table 1) the PCR products were stitched together in an overlap extension PCR. The resulting product was used for transformation of *S. gordonii* DL1.

For gene complementation, plasmid p*ssnA*_Comp_ was created by amplification of *ssnA* using SsnA.compF and SsnA.compR primers and genomic DNA from wild-type *S. gordonii* DL1 as template. SsnA.compF and SsnA.compR primers included *Eco*RI and *Bam*HI restriction enzyme sites, respectively. The amplicon was digested with *Bam*HI and *Eco*RI and ligated into pDL276 vector (Supplementary Table 1) that had also been digested with *Bam*HI and *Eco*RI (New England BioLabs). Successful construction of p*ssnA*_Comp_ was confirmed by sequencing and was used to transform *S. gordonii* Δ*ssnA* mutant to generate the genetically complemented strain *S. gordonii ssnA*_Comp_.

Plasmid p*ccpA*_Comp_ was generated by replacing the *arcR* gene in p*arcR*_Comp_^71^ with the *ccpA* gene. Primers CcpA_compF and CcpA_compR were used to amplify the *ccpA* gene using wild-type *S. gordonii* genomic DNA as template, generating a 1048-bp fragment. Lin-vecF and Lin-vecR primers were used to create linearized vector using p*arcR*_Comp_ plasmid as template, producing a 5797-bp linear vector. The In-Fusion HD PCR ligation cloning kit (Clontech Laboratories, Mountain View, California, USA) was employed to fuse the *ccpA* fragment to the vector backbone generating p*ccpA*_Comp_. The integrity of plasmid p*ccpA*_Comp_ was confirmed by sequencing and p*ccpA*_Comp_ was used for transformation of *S. gordonii ΔccpA* to generate *S. gordonii ccpA*_Comp_.

### Genetic manipulations of *E. coli*

Routine genetic manipulations were carried out using protocols described by Sambrook et al.^72^ and explained below. Plasmids and primers are described in Supplementary Table 1 and Supplementary Table 2.

### Expression and purification of recombinant SsnA

SsnA was cloned as a glutathione S-transferase (GST) fusion construct. The GST tag was cleaved where necessary. The *ssnA* gene, minus the C-terminal LPxTG cell anchor motif and N-terminal secretion signal domain, was amplified using primers ssnA_Pf7 and ssnA_Pr7, creating a product of 2150 bp. This product was cloned into pGEX-KT plasmid (Supplementary Table 1) and transformed into *E. coli* DH5α. For transformation, chemically competent *E. coli* cells were produced by culturing *E. coli* to early exponential phase (OD_600 nm_ ~ 0.3) in 50 mL LB, harvesting for 5 min at 5000 rpm and suspending the pellet in 20 mL ice cold 50 mM CaCl_2_. After standing in ice for 20 min, cells were harvested by centrifugation at 5000 rpm, 4 °C for 5 min and suspended in 4 mL ice cold CaCl_2_. Samples were distributed into 300 µL portions, DNA was added and incubated on ice for 40 min. Cells were heat-shocked at 42 °C for 2 min and plunged into ice. Transformants were recovered by adding 500 µL pre-warmed SOC medium containing 20 g L^−1^ Bacto tryptone, 5 g L^−1^ yeast extract, 10 mM NaCl, 2.5 mM KCl, 10 mM MgCl_2_, 10 mM MgSO_4_ and 20 mM glucose, and incubating for 1 h at 37 °C, 250 rpm before spreading across selective LB agar plates. pGEX-*ssnA* plasmid then was transferred to *E. coli* BL21 (DE3) pLysS (Stratagene, La Jolla, California, USA). Expression of GST-SsnA was induced by 1 mM isopropyl β-D-1-thiogalactopyranoside (Sigma Aldrich) for 4 h at 37 °C. A glutathione Sepharose column was used to elute GST-SsnA in 10 mM glutathione, 50 mM potassium phosphate, pH 7.2, 1 mM DTT (Sigma Aldrich). SDS-PAGE and Coomassie Brilliant Blue staining and zymography were performed to assess the size and activity of purified GST-SsnA.

### Cleavage of the GST tag from SsnA

The N-terminal 26 kDa GST protein tag was cleaved by thrombin. Purified SsnA was exchanged from elution buffer to TNC buffer (20 mM Tris-HCl [pH 7.5], 50 mM NaCl, and 1 mM CaCl_2_) for optimal cleavage of the GST-tag. Samples were incubated for 24 h at room temperature with 1 U bovine thrombin (Sigma) for every 0.025 mg of SsnA. Thrombin was inactivated by the addition of 0.3 mM phenylmethylsulfonyl fluoride (PMSF) and incubated at 37 °C, 15 min.

### Biofilm growth in the BioFlux 1000 microfluidic system

For mixed-species microcosm biofilm growth, saliva was collected from healthy volunteers who had not eaten or drunk (except water) in the last hour and had not taken antibiotics in the last 3 weeks. Ethical approval for the collection of saliva was granted by the Newcastle University Faculty of Medical Sciences Ethics Committee (ref. 1853/519/2020). Where needed, saliva was treated with dithiothreitol, precipitates were removed by centrifugation, and saliva was sterilised by filtration to create cell-free saliva (CFS)^73^. The BioFlux 1000 (Fluxion, San Francisco, CA) was employed to grow mixed-species oral microcosm biofilms. Prior to inoculation, channels of BioFlux dual-flow 24 well plates (Fluxion) were primed by addition of 200 μL of cell-free saliva (CFS) and incubation at room temperature for 20 min. Excess CFS was then removed from the outlet well and replaced with 100 μL of untreated saliva. Flow was initiated from the outlet to inlet at 1.0 dyn/cm^2^ for 6 s. The plate was then incubated at 37 °C for 40 min to allow for seeding to take place. Each of the inlet wells was then filled with pre-warmed (at 37 °C) CFS and flow was initiated at 0.5 dyn/cm^2^ at 37 °C for 18 h and images were taken every 10 min. Where required, CFS in the inlet wells was supplemented with 2% sucrose or DNase enzymes. DNase enzymes used were bovine DNase I (Sigma Aldrich), *Bacillus licheniformis* NucB^67^ and *S. gordonii* SsnA. They were added to the inlets at a concentration of 5 μM. To visualise eDNA, 2.4 nM Yoyo-1 (LifeTechnologies) dye was included with CFS in both inlet wells.

### Imaging biofilms

Imaging of biofilms grown in the BioFlux 1000 was carried out using the BioFlux 1000Z Imaging Workstation consisting of a Zeiss Axio Observer Z1 microscope fitted with an environmental enclosure. A Hamamatsu camera (ORCA-Flash 4.0 LT: 4.2 megapixels with 6.5 micron pixels) and BioFlux Montage Software (Fluxion) were used for acquisition of images. Images were analysed using ImageJ v.1.48 (National Institutes of Health)^74^.

### Image analysis of biofilms cultured in the BioFlux

Time-lapse images of each channel were converted into a stack. Each stack was auto-thresholded using the ‘Otsu’ algorithm. A rectangle of w = 1000 h = 200 pixels (stock image size 1024 × 1024 pixels) was selected and saved as a region of interest (ROI). The average pixel intensity (mean grey value) was measured for each slice and plotted using SigmaPlot 13.0 software (Systat Software, San Jose, CA, USA).

### Visualisation of static biofilms

For visualisation of static biofilms with confocal laser scanning microscopy (CLSM), stained coverslips were rinsed with PBS and inverted onto a PBS-filled rubber frame secured on a microscope slide. Imaging was performed using a Nikon A1R confocal laser scanning microscope fitted with CFI PLAN APO VC objective (Nikon 60x/1.40 Oil). Images were captured with NIS-Elements C (v4.4, Nikon) software and processed using Imaris (v8.2, Bitplane) software.

### DNase test agar

The extracellular DNase activity of *S. gordonii* was assessed using DNase Test agar (Sigma Aldrich, Gillingham, UK). Bacterial cultures were streaked or spotted (10 µL) on DNase Test agar and incubated aerobically for 48 h at 37 °C. The DNA in the DNase Test agar was precipitated by flooding the plate with 1 N HCl.

### DNase activity assay of bacterial cultures

To determine the nuclease activity of bacterial cultures, overnight cultures were washed twice with PBS at pH 7.1 and resuspended in PBS to an optical density, OD_600 nm_ = 1. The DNase activity was then measured for 25 µL of the cell suspension using the Synergy HT microplate reader (BioTek). For experiments to assay the recovery of DNase activity following low-pH incubation, overnight cultures of *S. gordonii* DL1 cells grown in THYE medium at 37 °C were harvested and re-suspended in a pH 4.5 buffer (77.1 g/L ammonium acetate, 70 mL/L glacial acetic acid), a pH 5.5 buffer (96.3 mL of solution I [13.61 g/L KH_2_PO_4_ (BDH)] and 3.6 mL of solution II [35.81 g/L Na_2_HPO_4_]) and PBS (pH 7.1). Cells were incubated at room temperature for up to 2 h, washed in PBS, resuspended in fresh PBS and 25 µL aliquots were assayed for DNase activity using the fluorescence-based DNase activity assay. After 2 h, cells were harvested and washed in PBS at pH 7.1. Cultures that had been incubated in acidic buffers (pH 4.5 or 5.5) were split into equal portions prior to harvesting. One portion was resuspended in PBS while the other was resuspended in PBS containing 12.5 µg/mL chloramphenicol to inhibit *de novo* synthesis of proteins^75^. Cultures were incubated for up to a further 2 h before DNase activity was measured.

When investigating the activity of purified SsnA in the presence of metals, 40 µM EDTA was included in the reactions to chelate residual metal contaminants of the buffer or associated with the DNA substrate prior to addition of the putative metal co-factor. When this assay was employed to determine the optimal pH for SsnA enzyme activity, the following buffers were used: Sodium acetate trihydrate buffer (pH 4.5-5.5), Bis-Tris (pH 6.0-6.5), Tris-HCl (pH 7.0-9.0) and 3-Cyclohexylamino-1-propanesulfonic acid buffer (pH 9.7-11.0) (Sigma Aldrich).

### DNase zymography

Zymography was used to assess the activity of purified SsnA with or without the GST tag against double stranded DNA. Enzymes in Laemmli’s buffer were loaded (without boiling) onto 12% SDS-PAGE gels containing 100 μg/mL salmon sperm DNA (Sigma Aldrich). Following electrophoresis, the gel was incubated in an enzyme reactivation buffer (40 mM Tris-HCl, 5 mM CaCl_2_, 5 mM MgCl_2_ and 3% delipidated milk powder [Premier International Foods, St. Albans, UK]) for 20 h at 37 °C. The gel was stained with ethidium bromide (0.5 μg mL^−1^) for 30 min and washed 3 times for 15 min each, in distilled water. The gel was visualised under ultraviolet light using G:BOX transilluminator (Syngene).

### RNA extraction and reverse transcription

Four mL of cell culture was mixed with 4 mL RNAlater (Life Technologies), and the tubes were vortex-mixed for 5 s and incubated at 20 °C for 5 min. Following incubation, cells were harvested at 3800 *g* for 15 min at 20 °C, the supernatant was discarded, and the pellets were frozen at −80 °C for no longer than 72 h and RNA was extracted^76^. Samples were thawed at 20 °C and resuspended in 100 μL spheroplasting buffer plus mutanolysin at 500 U/mL. Cells were incubated at 37 °C for 5 min. Total RNA was extracted using the Ambion RiboPure Bacteria RNA Purification kit (Life Technologies) according to the manufacturer’s instructions. RNA concentrations were determined using a NanoDrop ND‐1000 Spectrophotometer (Nanodrop Technologies, LLC, Wilmington, DE, USA). The RNA was analysed by gel electrophoresis to ensure the integrity of the RNA. One μg of total RNA from bacterial cells was reverse transcribed using the QuantiTect Reverse Transcription kit (Qiagen, Hilden, Germany) in accordance with the manufacturer’s instructions.

### RT-qPCR analysis of *ssnA* expression

RT-qPCR reaction mixtures were prepared containing 0.25 μL of cDNA samples and QuantiTect SYBR Green mix (Qiagen), 1 μM forward primer qRT-ssnAF and qRT-ssnAR reverse primer (Supplementary Table 2) in a total volume of 20 μL. Reactions were performed in triplicate using QuantStudio 3 (ThermoFisher Scientific, Waltham, Massachusetts, USA) with the following thermocycling program: initial denaturation at 95 °C for 2 min and 40 cycles of amplification at 95 °C for 15 s, 55 °C for 10 s and 72 °C for 30 s. The Comparative CT Method (ΔΔCT) was used to analyze RT-qPCR data, and data were normalized to the 16 S rRNA gene as a reference (Supplementary Table 2, qRT-16S-F and qRT-16S-F). RT-qPCR reactions were validated using melt curve analysis.

### Statistical analyses

All graphs were plotted and statistical tests were performed using GraphPad Prism (GraphPad Software, La Jolla California, USA) version 9. Details of statistical tests are given in figure legends.

### Reporting summary

Further information on research design is available in the Nature Research Reporting Summary linked to this article.

## Supplementary information

Supplementary Material

Reporting Summary

Supplementary Movie 1

Supplementary Movie 2

Supplementary Movie 3

Supplementary Movie 4

## Data Availability

Quantitative data underpinning the figures in the main manuscript are available at the Newcastle University Research Repository (10.25405/data.ncl.21539340). Other data are available from the corresponding author upon reasonable request.
