# Supplementary material for: Interspecies competition in oral biofilms mediated by Streptococcus gordonii extracellular deoxyribonuclease SsnA
Source: NPJ Biofilms Microbiomes. 2022 Dec 12;8:96. doi: 10.1038/s41522-022-00359-z (PMC9744736; doi:10.1038/s41522-022-00359-z)
Supplement: Supplementary file 1 — Supplementary Material [file 41522_2022_359_MOESM1_ESM.pdf]

Interspecies competition in oral biofilms mediated by *Streptococcus gordonii* extracellular deoxyribonuclease, SsnA

Nadia Rostami, Robert C. Shields, Hannah J. Serrage, Catherine Lawler, Sufian Yassin, Halah Ahmed, Achim Treumann, Paul Thompson, Kevin J. Waldron, Angela H. Nobbs, and Nicholas S. Jakubovics

Supplementary Information:

2-10: Supplementary Figures 1-8.

10-12: Supplementary Tables 1-2.

13: Supplementary Method.

14: Supplementary References.

Supplementary Figures

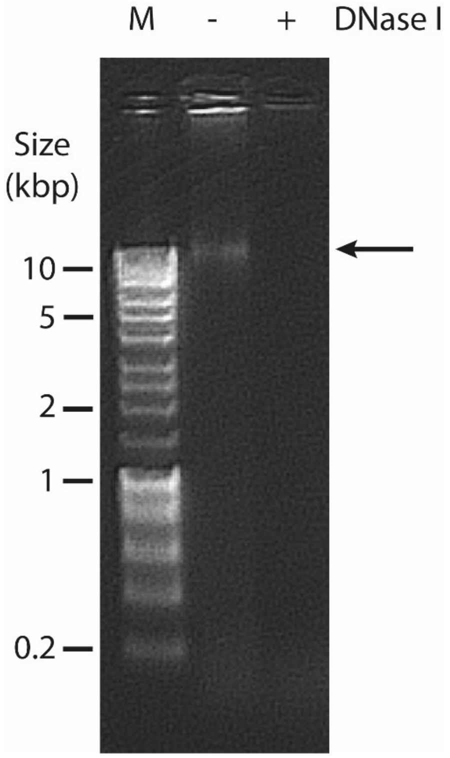

Supplementary Figure 1. Digestion of *S .mutans* GS5 eDNA with DNase I. Biofilms were grown for 72 h and eDNA was extracted. A portion of eDNA was incubated with 500 µg/mL DNase I for 2 h at 37°C and was run on an agarose gel alongside untreated eDNA. The arrow indicates the major band of eDNA (>10 kbp) that was present only in the untreated preparation.

a

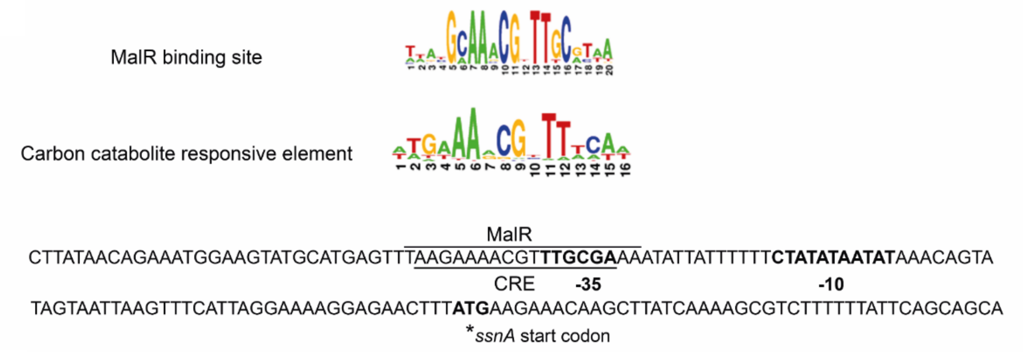

b

	SsnA enz	Wild type	ΔssnA	ssnA$_{Comp}$	ΔccpA	ccpA$_{Comp}$	ΔmalR
No sugar	94 ± 3	53 ± 3	0	45 ± 11	51 ± 4	50 ± 17	46 ± 14
Glucose	73 ± 6	0	0	0	43 ± 3	0	0
Maltose	69 ± 11	0	0	0	36 ± 13	18 ± 16	0
Sucrose	91 ± 5	0	0	0	54 ± 8	0	0
Galactose	62 ± 18	50 ± 6	0	50 ± 5	39 ± 8	48 ± 14	53 ± 17

0 — 100

Supplementary Figure 2. Regulation of ssnA by sugars. (a) *ssnA* promoter region. Putative binding sites for MalR or CcpA identified in the promoter region of the *ssnA* gene are shown as sequence logos. (b) Wild type *S. gordonii*, Δ*ccpA, ccpA*$_{Comp}$, Δ*malR* were grown on DNase Test Agar supplemented with 2% (w/v) sugars for 48 h prior to precipitation of DNA with 1N HCl. Purified SsnA (5 µg/mL; 'SsnA enz') and *S. gordonii* Δ*ssnA* were included as positive control and negative control, respectively. To obtain % DNA degradation, images were converted to binary and the white to black ratio was determined using ImageJ v.1.48 software. The mean ± standard deviation is shown for 4 independent experiments. *S. gordonii* DNase activity was strongly inhibited by all sugars but galactose. Inhibition was alleviated in the Δ*ccpA* mutant and restored in *S. gordonii ccpA*$_{Comp}$.

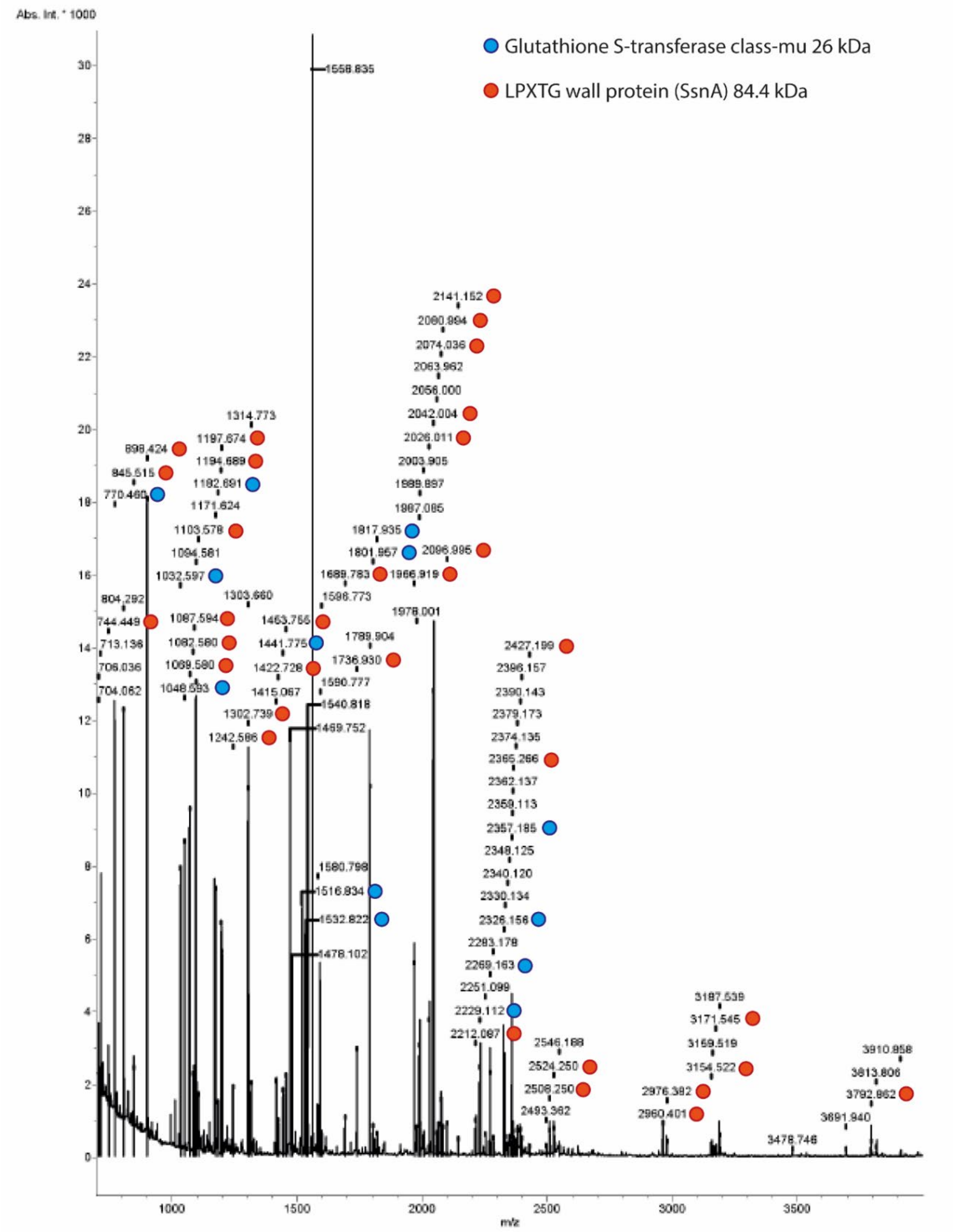

Supplementary Figure 3. Peptide mass fingerprinting of purified SsnA-GST fusion construct. The protein was extracted from an SDS-PAGE gel, digested with trypsin and peptides were analysed by MALDI TOF/TOF. Peptides matching fragments of GST and SsnA are indicated. No other proteins were identified.

a b

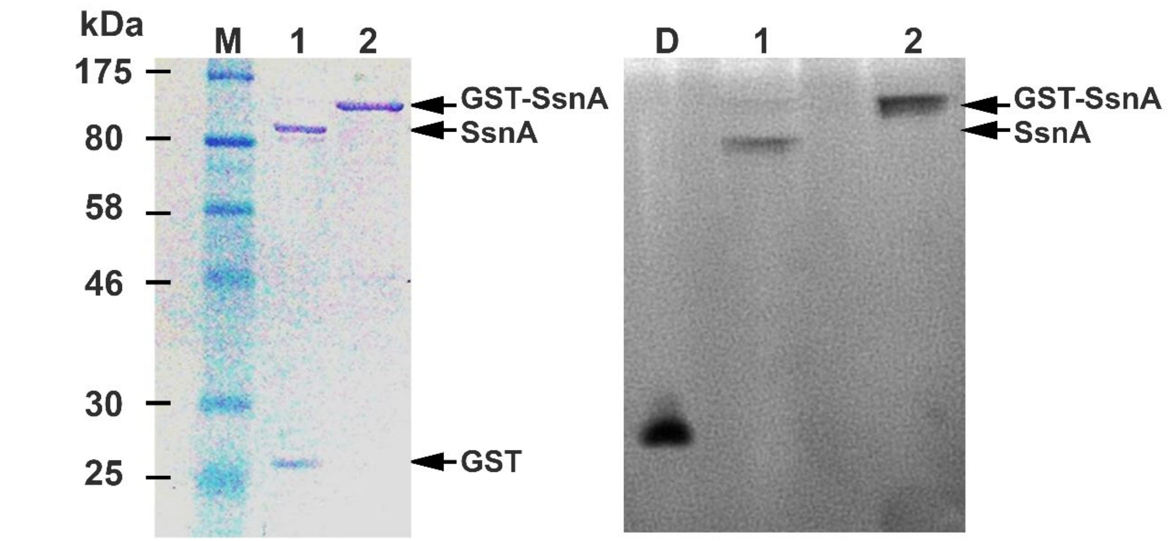

Supplementary Figure 4. GST-tagged SsnA is active against double stranded DNA. (a) Coomassie Brilliant Blue staining of SDS-PAGE gel of **1** thrombin cleavage products, GST (26 kDa) and SsnA (85 kDa) and **2** intact GST-SsnA (111 kDa). (b) In gel zymography of GST-SsnA DNase activity (111 kDa), SsnA (85 kDa), and DNase I (29 kDa) were run on a 12% SDS-PAGE gel containing dsDNA. Extracellular nucleases were reactivated, and gels were stained with ethidium bromide and visualised with ultraviolet light. The colours were inverted for clarity, the dark zones indicate areas of DNA digestion by **D-**DNase I, **1-** SsnA and **2-** GST-SsnA.

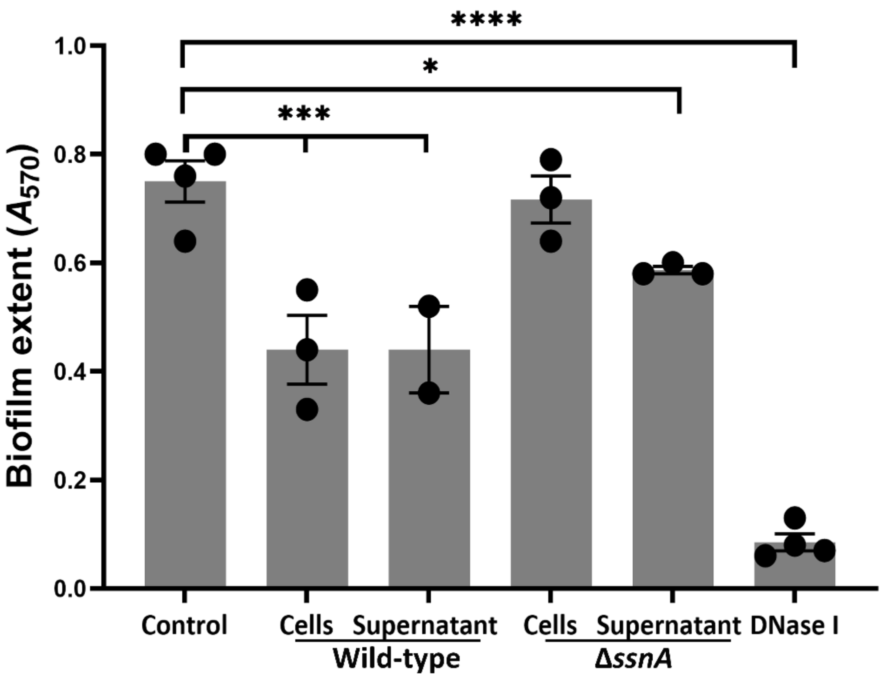

Supplementary Figure 5. *Streptococcus mutans* GS-5 biofilm disruption by *Streptococcus gordonii*. Biofilm extent of pre-established *Streptococcus mutans* GS-5 biofilms grown anaerobically for 20 h treated with *S. gordonii* cell fraction or spent medium for 1 h at 37°C. The total amount of remaining biofilm was determined using the crystal violet assay. DNase I (5 µg/mL) was included as positive control. Bars indicate mean values from 4 independent experiments and SE is shown. The distribution of data passed the Shapiro-Wilk normality test. One-way ANOVA and Dunnett's multiple comparisons were performed using GraphPad prism 9 to determine statistical significance. * $p < 0.05$; *** $p < 0.001$; **** $p < 0.0001$.

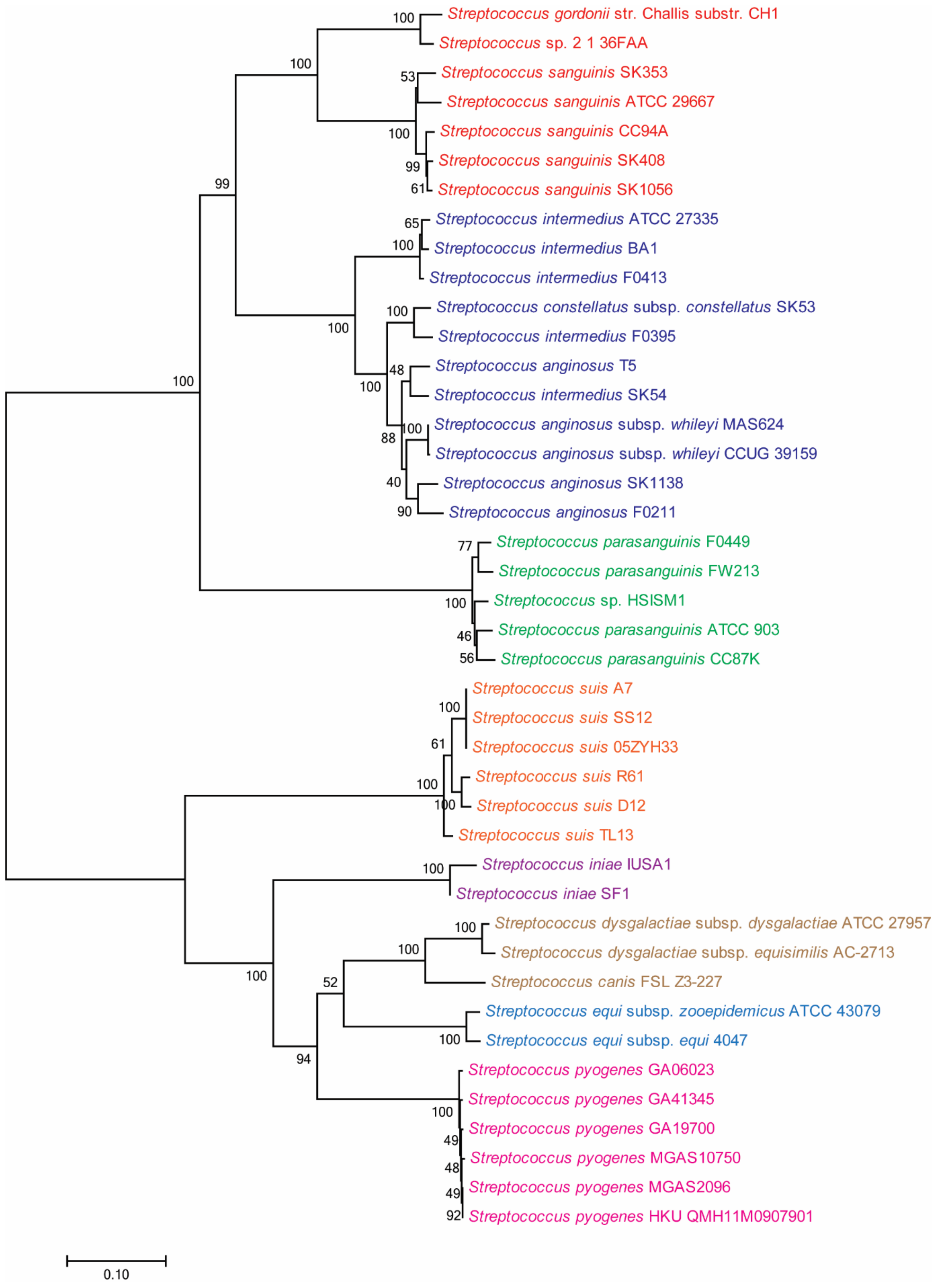

Supplementary Figure 6. Homologues of *S. gordonii* SsnA. A search of the OrthoDB database (https://www.orthodb.org/) for SsnA from *S. gordonii* Challis (Accession A8AYR8)

identified 47 homologues in streptococci. After removing 5 incomplete sequences, the remaining protein sequences were aligned with CLUSTALW in MEGA 7[1] . A neighbor-joining tree was constructed in MEGA and drawn using the ETE Toolkit (http://etetoolkit.org/). At each branch point, the percentage of trees with the branch structure shown is indicated from 500 bootstrap replicates. The scale bar represents 0.1 substitutions per nucleotide position. Closely related homologues are shown in the same colour.

a b

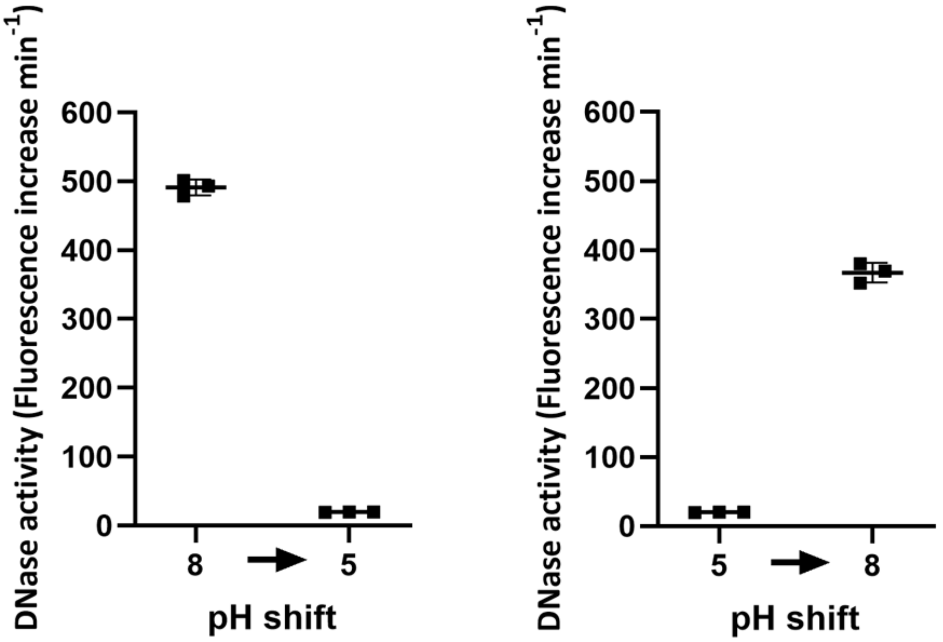

Supplementary Figure 7. Acid inhibition of recombinant SsnA is reversible upon incubation at a high pH. (a) The nuclease activity of recombinant SsnA was measured using the quantitative fluorescent assay, at pH 8 (Tris-HCl buffer). The pH of the reaction was then adjusted to pH 5 by addition of an acidic buffer (Sodium acetate trihydrate buffer pH 3.7) and incubated at room temperature for 15 min before the enzyme activity was measured again.(b) The nuclease activity of recombinant SsnA was measured at pH 5 (Sodium acetate trihydrate buffer pH 5), the pH of the reaction was then adjusted to pH 8 by addition of a basic buffer (3-Cyclohexylamino-1-propanesulfonic acid buffer pH 11) and incubated at room temperature for 15 min before the enzyme activity was measured again.

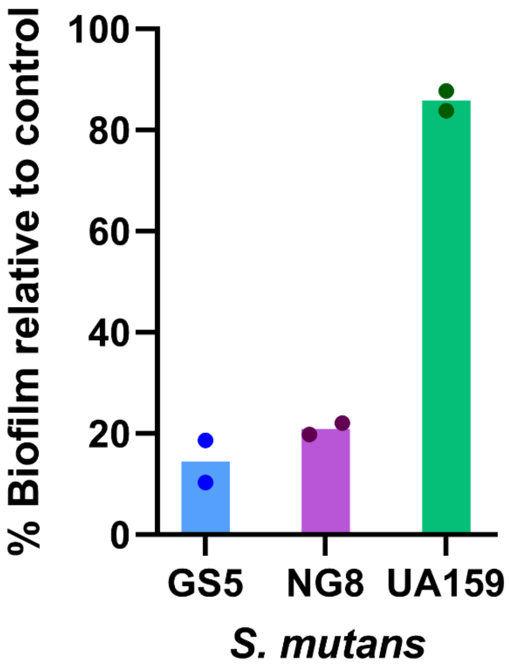

Supplementary Figure 8. Effect of NucB on biofilm formation by different strains of *S. mutans*. Biofilms were formed for 20 h in microtitre plate wells in the presence or absence of NucB (3 µg/mL). Following washing, residual biofilm biomass was detected by crystal violet staining. The percentage of biomass relative to the control is shown (mean and data points; n = 2).

Supplementary Tables

Supplementary Table 1. Strains and plasmids

**Strain or plasmid**	**Description**	**Source or reference**
***S. gordonii* strains**		
DL1	Wild-type	NCTC 7868
Δ*ssnA*	*ssnA*::*ermAM*	This study
Δ*ccpA*	*ccpA*:: *aphA3*	This study
Δ*malR*	*malR*::*ermAM*	This study
$ssnA_{\text{Comp}}$	*ssnA*::*ermAM*/p$ssnA_{\text{Comp}}$	This study
$ccpA_{\text{Comp}}$	*ccpA*:: *aphA3*/p$ccpA_{\text{Comp}}$	This study
S. mutans strains		
GS-5	Wild-type	[2]
S. aureus FH7		[3]
E. coli strains		
DH5α	F– Φ80*lac*ZΔM15 Δ(*lac*ZYA-*arg*F) U169 *rec*A1 *end*A1 *hsd*R17 (rK–, mK+) *pho*A *sup*E44 λ– *thi*-1 *gyr*A96 *rel*A1	Invitrogen
BL21(DE3)pLysS	F^- *ompT* $hsdS_B(r_B^-\ m_B^-)$ *gal dcm* (DE3) pLysS (*CamR*)	Stratagene
**Plasmids**		
pDL276	*Ori*, *lacZa*, *KanR*	[4]
p$ssnA_{\text{Comp}}$	P_{ssnA}-*ssnA*::*kanR*	This study
p$arcR_{\text{Comp}}$	P_{CP25}-*arcR::ermR*	[5]
p$ccpA_{\text{Comp}}$	P_{CP25}-*ccpA::ermR*	This study
pGEX-KT	pMB1 *ori*, *lacIq*, *lacZ*, P_{tac} , *gst*,*ampR*	[6]
pGEX-*ssnA*	P_{tac}-*gst-ssnA*	This study
pFW5		[7]
pK18	*lacZα*, *kanR*	[8]
pVA838	*ermR*, *tcR*, *camR*	[9]

Supplementary Table 2. Primers (5'-> 3')

SsnAF1	TTTATCAGAAATTGATTGCC
SsnAR1	TCATAAAGTTCTCCTTTTCCTA
SsnAF2	TAACCTAGAGTAAGCTCTAAACATC
SsnAR2	TGTCAAAGCTACCAGTACTTG
aad9_SsnAF	AGGAGAACTTTATGAATACATACGAACAAATTAATA
aad9_SsnAR	GCTTACTCTCTAGGTTATAATTTTTTTAATCTGTTATTTAA
SsnA.compF	CCGGAATTCTTGCAGTGAGGAAAGTTG
SsnA.compR	CGCGGATCCCTTCAGCTCAAGAGGCAG
CcpAF1	CAAACCGTGGTATTTAGTTAAAATTAAGTTGTCAAATTCA
CcpAR1	AATCCATCTTGTTCAATCATATTGCTTCCTTTCTTAAAGTTGAAAATAACGTT
KanR_F	ACTTTAAGAAAGGAAGCAATATGATTGAACAAGATGGATTGCACG
KanR_R	CCAAATGGTGACTTTATCTTTCAGAAGAACTCGTCAAGAAGGCGA
CcpAF2	TTCTTGACGAGTTCTTCTGAAAGATAAAGTCACCATTTGGTGGCT
CcpAR2	AGCTTTTTAGAATACTCTGACGGTGTAATAACAAAG
CcpA_compF	CCTGGGGTAATGACTCTCTAGCTTGA
CcpA_compR	AGTCATTACCCCAGGTTATTTTCTAGTTGAGTTCCGTTCGTAAATACCATGAG
Lin-vecF	GGGCTGGTATAATAAGAAAGGAAGCAATATGAACACAGACGA
Lin-vecR	TTATTATACCAGCCCCCTCACTACATGT
malRF1	AATTGGCTGCCATTCGTTAC
malRR1	GTTCATGTAATCACTCCTTACAGTTGAGGGCGAAACC
malRF2	ACGGGAGGAAATAATTCTGGCATCTGGTGTTTGTGAT
malRR2	ACCATCAGAGGCTGTCGGTA
ermAMF2	GAATTATTTCCTCCCGTTAA
ermAMR2	GGAGTGATTACATGAACAAA
ssnA_Pf7	GACTGGATCCGAAGAGACGGAAAATTCTTC
ssnA_Pr7	CGATGAATTCACTCTTTTTGTTTTCACCTGA
qRT-ssnAF	CAG GCCTCC ATCGACTAAATC
qRT-ssnAR	GTGACGTAGTTGGAGTGGTTAG
qRT-16S-F	AGACACGGCCCAGACTCCTAC
qRT-16S-R	CTCACACCCGTTCTTCTCTTACAA

Supplementary Method

Peptide Mass Fingerprinting

To confirm the identity of the SsnA-GST fusion protein construct, samples were analysed by peptide mass fingerprinting. All sample handling was performed with fresh buffers, new containers and wearing gloves to minimise contamination. Six samples (3 µg each) were separated by SDS-PAGE. The gel was transported to the Newcastle University Protein and Proteome Analysis (NUPPA) facility, where relevant bands were extracted from the gel, digested with trypsin, and analysed using a matrix-assisted laser desorption/ionization time of flight/time of flight (MALDI TOF/TOF) mass spectrometer. Peptide matches were searched against the known protein sequences of *S. gordonii* using BioTools version 3.1.

Supplementary References

1 Kumar, S., Stecher, G. & Tamura, K. MEGA7: Molecular Evolutionary Genetics Analysis Version 7.0 for Bigger Datasets. *Mol Biol Evol* **33**, 1870-1874, doi:10.1093/molbev/msw054 (2016).

2 Biswas, S. & Biswas, I. Complete genome sequence of *Streptococcus mutans* GS-5, a serotype c strain. *J Bacteriol* **194**, 4787-4788, doi:10.1128/JB.01106-12 (2012).

3 Shields, R. C. *et al.* Efficacy of a marine bacterial nuclease against biofilm forming microorganisms isolated from chronic rhinosinusitis. *PLOS ONE* **8**, e55339, doi:10.1371/journal.pone.0055339 (2013).

4 Dunny, G. M., Lee, L. N. & LeBlanc, D. J. Improved electroporation and cloning vector system for gram-positive bacteria. *Appl Environ Microbiol* **57**, 1194-1201, doi:10.1128/aem.57.4.1194-1201.1991 (1991).

5 Robinson, J. C. *et al.* ArcR modulates biofilm formation in the dental plaque colonizer *Streptococcus gordonii*. *Mol Oral Microbiol* **33**, 143-154, doi:https://doi.org/10.1111/omi.12207 (2018).

6 Hakes, D. J. & Dixon, J. E. New vectors for high level expression of recombinant proteins in bacteria. *Anal Biochem* **202**, 293-298, doi:https://doi.org/10.1016/0003-2697(92)90108-J (1992).

7 Vos, T. *et al.* Global, regional, and national incidence, prevalence, and years lived with disability for 328 diseases and injuries for 195 countries, 1990–2016: a systematic analysis for the Global Burden of Disease Study 2016. *The Lancet* **390**, 1211-1259, doi:https://doi.org/10.1016/S0140-6736(17)32154-2 (2017).

8 Schäfer, A. *et al.* Small mobilizable multi-purpose cloning vectors derived from the *Escherichia coli* plasmids pK18 and pK19: selection of defined deletions in the chromosome of *Corynebacterium glutamicum*. *Gene* **145**, 69-73, doi:https://doi.org/10.1016/0378-1119(94)90324-7 (1994).

9 Macrina, F. L., Tobian, J. A., Jones, K. R., Evans, R. P. & Clewell, D. B. A cloning vector able to replicate in *Escherichia coli* and *Streptococcus sanguis*. *Gene* **19**, 345-353, doi:10.1016/0378-1119(82)90025-7 (1982).
